# A DNase from a Fungal Phytopathogen Is a Virulence Factor Likely Deployed as Counter Defense against Host-Secreted Extracellular DNA

**DOI:** 10.1128/mBio.02805-18

**Published:** 2019-03-05

**Authors:** Hee-Jin Park, Weiwei Wang, Gilberto Curlango-Rivera, Zhongguo Xiong, Zeran Lin, David A. Huskey, Martha C. Hawes, Hans D. VanEtten, B. Gillian Turgeon

**Affiliations:** a Plant Pathology & Plant-Microbe Biology Section, School of Integrative Plant Science, Cornell University, Ithaca, New York, USA; b College of Plant Sciences, Jilin University, Changchun, China; c Institute of Tropical Agriculture and Forestry, Hainan University, Haikou, China; d Department of Soil, Water and Environmental Sciences, University of Arizona, Tucson, Arizona, USA; e School of Plant Science, Bio5 Institute, University of Arizona, Tucson, Arizona, USA; f Division of Plant Pathology & Microbiology, The Department of Plant Sciences, College of Agriculture and Life Sciences, Tucson, Arizona, USA; University of Melbourne; Auburn University; Pacific Northwest National Laboratory

**Keywords:** NETs, extracellular DNA, fungi, virulence determinants

## Abstract

Histone-linked extracellular DNA (exDNA) is a component of neutrophil extracellular traps (NETs). NETs have been shown to play a role in immune response to bacteria, fungi, viruses, and protozoan parasites. Mutation of genes encoding group A Streptococcus extracellular DNases (exDNases) results in reduced virulence in animals, a finding that implies that exDNases are deployed as counter defense against host DNA-containing NETs. Is the exDNA/exDNase mechanism also relevant to plants and their pathogens? It has been demonstrated previously that exDNA is a component of a matrix secreted from plant root caps and that plants also carry out an extracellular trapping process. Treatment with DNase I destroys root tip resistance to infection by fungi, the most abundant plant pathogens. We show that the absence of a single gene encoding a candidate exDNase results in significantly reduced virulence of a fungal plant pathogen to its host on leaves, the known infection site, and on roots. Mg^2+^-dependent exDNase activity was demonstrated in fungal culture filtrates and induced when host leaf material was present. It is speculated that the enzyme functions to degrade plant-secreted DNA, a component of a complex matrix akin to neutrophil extracellular traps of animals.

## Abstract (precis)

We document that the absence of a single gene encoding a DNase in a fungal plant pathogen results in significantly reduced virulence to a plant host. We compared a wild-type strain of the maize pathogen Cochliobolus heterostrophus and an isogenic mutant lacking a candidate secreted DNase-encoding gene and demonstrated that the mutant is reduced in virulence on leaves and on roots. There are no previous reports of deletion of such a gene from either an animal or plant fungal pathogen accompanied by comparative assays of mutants and wild type for alterations in virulence. We observed DNase activity, in fungal culture filtrates, that is Mg^2+^ dependent and induced when plant host leaf material is present. Our findings demonstrate not only that fungi use extracellular DNases (exDNases) for virulence, but also that the relevant molecules are deployed in above-ground leaves as well as below-ground plant tissues. Overall, these data provide support for a common defense/counter defense virulence mechanism used by animals, plants, and their fungal and bacterial pathogens and suggest that components of the mechanism might be novel targets for the control of plant disease.

## INTRODUCTION

Histone-linked extracellular DNA (exDNA) is an integral component of neutrophil extracellular traps (NETs), a complex matrix of DNA and proteins that ensnares menacing pathogens and thus plays a critical role in cellular defense in animals (1–6). If NET exDNA is degraded by the addition of DNase I, the capacity to immobilize and kill invading pathogens is lost (1, 5), demonstrating that DNA macromolecules are essential to NET trapping. As an offset measure, extracellular DNases (exDNases) produced by microbial pathogens of animals have been shown to be key factors in microbial counter defense (7–9). For example, mutation of exDNase-encoding genes in Streptococcus and Staphylococcus spp. results in augmented trapping and reduced spread in the host (3, 10, 11). Extracellular trapping is not exclusive to neutrophils and has now been documented for diverse animal tissues and organs, and it was shown to play a role in disease response to a variety of infectious agents, including bacteria, fungi, viruses, and protozoan parasites (12–15).

The relevance of the exDNA/exDNase mechanism to plants and their pathogens is unknown. Histone-linked exDNA has been recognized as a component of the extracellular matrix secreted from root caps of pea, corn, cotton, and soybean that, along with root border cells, traps soilborne bacteria, fungi, and heavy metals (16). As with neutrophils, treatment of pea root tips and border cells with DNase I abolishes trapping of certain fungi and the normal resistance to infection and results in 100% root rot (17, 18). To our knowledge, there is only one report that tackles this issue using strains carrying loss-of-function mutations in microbial candidate exDNase-encoding genes. When two candidate bacterial exDNase genes, *nucA* and *nucB,* of the tomato pathogen Ralstonia solanacearum were deleted, the resulting mutant was trapped by the exDNA matrix of tomato root border cells *in vitro* and was reduced in systemic dispersal and virulence *in vivo* compared to the wild-type strain (19). This work with a bacterial plant pathogen confirmed, for the first time with any plant pathogen, that exDNases are indeed virulence factors involved in counter defense against host exDNA in plant-produced NETs.

Do the most abundant pathogens of plants, the fungi, also use exDNases as a counter defense against plant cell NETs? Numerous reports have definitively demonstrated that fungal pathogens of animals secrete DNases (14, 20–22), but relatively few reports have shown that fungal pathogens of plants or even saprobes secrete DNases (23, 24). Early work on the saprobe Neurospora crassa described mutants that were less able to degrade DNA in assays for nucleases (25); however, none of the corresponding genes is an ortholog of the exDNases described above or below. Work with the pea pathogen Nectria haematococca revealed that increased exDNase activity in strains with a dispensable chromosome carrying a putative DNase-encoding gene was correlated with increased virulence on pea plants (26). However, in no case has a fungal gene from either an animal or plant pathogen encoding a candidate secreted exDNase been disabled and the effect of its loss on virulence to the host been reported.

Herein, we document that the absence of a single gene encoding a DNase in a fungal plant pathogen results in significantly reduced virulence to a plant host. We compared a wild-type strain of the maize pathogen Cochliobolus heterostrophus and an isogenic mutant lacking a candidate secreted DNase-encoding gene and demonstrated that the mutant is reduced in virulence to its plant host on leaves, the known site of infection, and on roots. We show DNase activity in fungal culture filtrates that is Mg^2+^ dependent and induced in response to plant host leaf material. Our findings link the importance of secreted DNases to the virulence abilities of both animal and plant pathogens (17, 27, 28). The exDNase counter defense/exDNA trapping mechanism should be considered an important new target for controlling plant disease.

## RESULTS

### C. heterostrophus has many DNase-encoding genes.

When the C. heterostrophus strain C4 genome (http://genome.jgi.doe.gov/CocheC4_1/CocheC4_1.home.html) was searched for proteins annotated as DNases, 30 such genes were identified. BLAST searches with previously identified fungal DNases as a query did not reveal additional proteins beyond the 30 identified. There was no hit when the sequence from Fusarium solani f. sp. *phaseoli*, identified as a DNase by Hadwiger et al. (23), was used as a query. Furthermore, this protein does not have domains identifying it as a DNase. Five genes, named *NUC1* through *NUC5,* corresponding to a subset of the 30 proteins (Joint Genome Institute [JGI] protein identifiers [IDs] 144206, 149183, 33717, 122478, and 83474, respectively) with approximately 0.5 or higher neural network (NN) secretion scores as determined by SecretomeP (29) and with a clear DNase domain were chosen for deletion.

Nuc1, Nuc2, and Nuc3 are predicted TatD DNases and metallo-dependent hydrolases, while Nuc4 and Nuc5 are predicted endonuclease/exonuclease/phosphatases. Only Nuc2, Nuc4, and Nuc5 proteins have a secretion signal as determined by SignalP (30) (Table 1). Protein sizes vary from 314 to 613 amino acids.

**TABLE 1:** Candidate exDNase-encoding proteins and phenotypes of gene deletion strains

Protein	Annotation	NN	Phenotype^*a*^					
			SP	TM	G&P	Con	App	Vir
144206 (Nuc1)	TatD deoxyribonuclease (Mg^2+^ dependent)	0.456	−	−	(+)	+	+	<
149183 (Nuc2)	TatD deoxyribonuclease (Mg^2+^ dependent)	0.529	+	−	+	+	+	+
33717 (Nuc3)	TatD deoxyribonuclease	0.890	−	−	+	+	+	+
122478 (Nuc4)	DNase I-like, endonuclease/exonuclease/phosphatase	0.775	+	−	+	+	+	+
83474 (Nuc5)	DNase I-like, endonuclease/exonuclease/phosphatase	0.627	+	−	+	+	+	+

a NN score, reference 29; SP, SignalP (30); TM, transmembrane domain; G&P, growth and pigmentation; Con, conidiation; App, ability to form appressoria; Vir, virulence. Under phenotype, left two columns: +, presence of SP or TM; −, absence of SP or TM. Following columns: +, wild-type for G&P, Con, App, Vir; (+), slightly reduced growth; <, reduced virulence.

### DNase deletion mutants display wild-type or near-wild-type morphological phenotypes.

Strains with confirmed single-gene deletions are shown in Fig. S1A to C in the supplemental material. Strains with double deletions (lacking *NUC1* and *NUC2*) were confirmed by PCR, as shown in Fig. S1D. Complementation of the *nuc1* mutant 144206-2-1 was confirmed by PCR, as shown in Fig. S2.

10.1128/mBio.02805-18.1 FIG S1 Deletion strategy and diagnostic PCR reactions for five candidate C4 exDNase mutants and *nuc1 nuc2* double mutants. Primers used are listed in Table S1. (A) Strategy to delete DNase-encoding genes. Pairs of primers matching each DNase-encoding genes 5′ (FP1/RP1) and 3′ (FP2/RP2) flanking DNA were used to amplify the 5′ and 3′ DNA flanking each gene of interest. A third primer pair (hygB-F/hygB-R or nptII-F/nptII-R) was used to amplify the selectable marker (40). (B) Proof of deletion of DNase-encoding genes. The primer pair FP/RP is internal to each gene. A band is expected with WT DNA or DNA from an ectopic insertion of transforming DNA, but not with DNA from a targeted deletion mutant. Two or three candidate mutants were screened for each attempted gene deletion, and most were targeted deletions, as the WT band was missing. The primer pair UF/PtrpC was used to test correct insertion into the 5′ flank of each gene. UF is upstream of the flanking DNA used to delete the ORF, while PtrpC is a reverse primer located in the promoter of the *hygB* gene. A band is expected in the case of a correct insertion event but should be absent in WT and ectopic integrants. The primer pair DR/TtrpC was used to test correct insertion into the 3′ flanking end of each gene. DR is downstream of the flanking DNA used to delete the ORF, while TtrpC is a forward primer located in the terminator of the *hygB* gene. A band is expected in the case of a correct insertion event but should be absent in WT and ectopic integrants. To verify gene deletion and targeted insertion of the selectable marker into the native locus of each gene, a set of gene-specific primers internal to the deleted gene (FP/RP) and two sets of primer pairs in which one primer was internal to the introduced selectable marker and the other was external to either the 5′ (UF/PtrpC) or 3′ (DR/TtrpC) flanking region used to delete the gene were used (Table S1), as previously described. (C) PCR evidence of deletion of DNase-encoding genes. Primer strategy shown in panel B was used to verify desired mutants. The position of each candidate DNA is the same in panels A to C. Strains examined are indicated on the top line by nomenclature; e.g., 33717-1-1 is a mutant deleted for the *NUC3* gene encoding protein ID 33717, and 1-1 indicates single conidium 1 from mutant 1 of 33717. (A) Primers FP/RP. (B) Primers UF/PtrpC. (C) Primers DR/TtrpC. Verified mutants are indicated by asterisks, and a double asterisk indicates the 144206 mutant chosen for virulence and complementation studies. (D) PCR confirmation of *nuc1 nuc2* double-deletion mutants. Primers used are listed in Table S1. Eight independent candidate double mutants (*nuc1 nuc2* 1-1, 2-1, 4-2, 5-1, 8-2, 9-1, 11-2, and 12-1) were examined. Lanes 1 and 2 test for the *nuc1* gene, while lanes 3 and 4 test for the *nuc2* gene. In all cases, primer pair WW105/WW106 (FP/RP) was used in lane 1, primer pair WW103/BH13 (UF/pTrpC, see Fig. S2) was used in lane 2, primer pair WW113/WW114 (FP/RP) was used in lane 3, and primer pair WW111/BH13 (UF/pTrpC) was used in lane 4. Note that the progenitor strain mutant *nuc1* does not carry the *NUC1* gene and that all candidate double mutants lack this gene (lane 1). The progenitor *nuc1* mutant strain carries the *nuc2* gene, but all candidate double mutants lack this gene (lane 3). Lane 2 demonstrates that in all strains the selectable marker that replaced *NUC1* inserted correctly into the native *nuc1* locus. The presence of a band in all lane 4s except in the progenitor confirms that the *nptII* deletion construct inserted at the native *nuc2* locus. Download FIG S1, PDF file, 0.2 MB. Copyright © 2019 Park et al. 2019 Park et al. This content is distributed under the terms of the Creative Commons Attribution 4.0 International license.

10.1128/mBio.02805-18.2 FIG S2 Strategy and proof of complementation. (A) Strategy as described in the text and by Wang et al (40). (B to D) Complementation of the *nuc1* mutant with WT *NUC1*. PCR confirmation of *nuc1* complementation. Primers are used listed in Table S1. Ten independent transformants, *nuc1*[*NUC1*]-1, -2, -3, -4, -5, -6, -7, -8, -9, and -10, were tested. (a) Primer pair WW105/WW106 is internal to the *NUC1* gene. Note that all candidates carry the introduced WT *nuc1* ORF, as does the WT, while the progenitor strain *nuc1* mutant does not. (b) Primer pair WW269/PtrpC was used to test correct insertion of the complementation construct into the downstream flank of *NUC1*. WW269 is a forward primer in the flanking DNA downstream of the *NUC1* ORF. PtrpC is a reverse primer in the promoter of the *nptII* gene. This band is missing in the original mutant, as expected. The weaker band in the WT lane is a nonspecific amplicon. (c) Primer pair WW273/TtrpC was used to test correct insertion of the complementation construct 3′ of the *NUC1* 3′ flank. WW273 is outside the F3 flank used for complementation. TtrpC is a forward primer in the *nptII* terminator. This band is missing in the original mutant, as expected. Five strains appear to have the integrated construct, one of which, c144206-2 (asterisk), was used for virulence testing. The weaker band in the WT is an enigma nonspecific amplicon. The band at about 2.3 kb in the remaining mutants is predicted to result from amplification from the *hygB* cassette in the original mutant strain (top line A), which also has TtrpC, and primer WW273 in the F3 flanking DNA. Download FIG S2, PDF file, 0.3 MB. Copyright © 2019 Park et al. 2019 Park et al. This content is distributed under the terms of the Creative Commons Attribution 4.0 International license.

**FIG 1:**
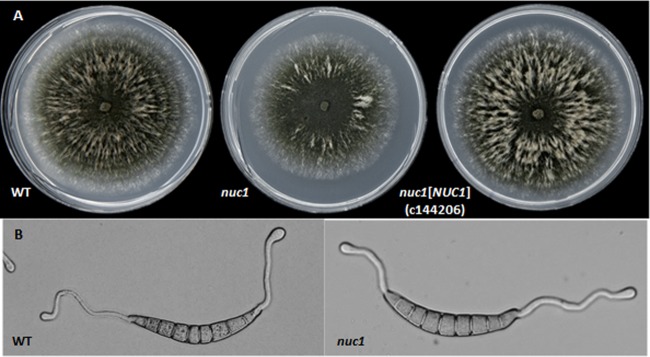
(A) Phenotype of WT C4, the *nuc1* mutant, and the complemented *nuc1* mutant (*nuc1*[*NUC1*]) grown on CMX medium under 16 h light/8 h dark for 7 days. Note the slight reduction in *nuc1* mutant growth compared to the WT. The complemented mutant was restored to WT growth. All other mutants grew like the WT (except the double mutant, which grew like the *nuc1* single mutant). (B) Equivalent germination and appressorium formation of WT and the *nuc1* mutant.

All mutants had morphological, asexual and sexual reproductive, and pigmentation characteristics similar to those of the wild type (WT) (Table 1). Mutants lacking *NUC1* grew slightly more slowly than WT (Fig. 1A, middle), as did the *nuc1 nuc2* double mutant (not shown). Complementation fully restored WT-level growth to mutants lacking *NUC1* (Fig. 1A, right). Conidial germination rate and ability to form appressoria were the same for WT, all mutants (Fig. 1B), and the *nuc1*[*NUC1*] complemented strains. Host penetration characteristics were like those of WT.

Thus, all traits important for successful early infection of the host were comparable for the mutants and WT.

### Strains lacking *NUC1* are reduced in virulence on both leaves and roots.

**FIG 2:**
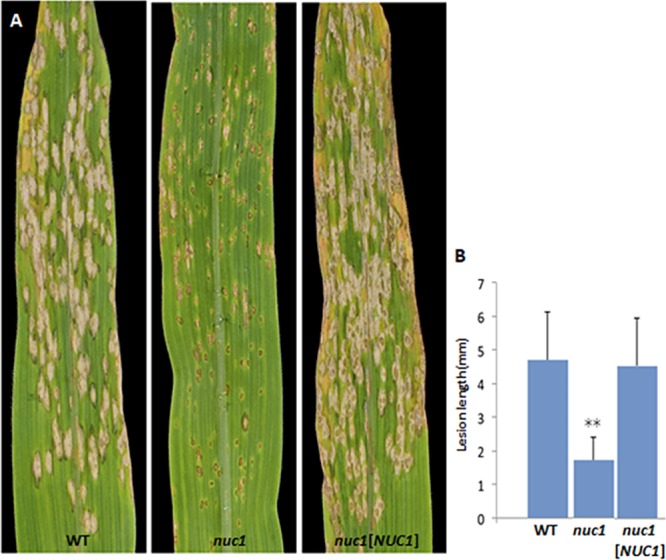
Virulence of the *nuc1* mutant is reduced on leaves. (A) Spray inoculation of WT C4, the *nuc1* mutant, and the complemented *nuc1* mutant on leaves of corn cultivar of W64A-N. The *nuc1* mutant is greatly reduced in virulence. (B) Lesion length comparisons from panel A. Error bars are the standard deviation. Double asterisks represent a *P* value of <0.01 in *t* test analysis in which the mutant and the complemented strain were compared with the WT strain.

Of the five genes deleted in WT strain C4, the lack of one (*NUC1*, Table 1 and Fig. S1C) resulted in a reduced virulence phenotype on maize both in spray inoculation of leaves and root inoculation assays. On leaves, lesions were reduced in size compared to the WT, while lesions produced by complemented strains were the same as the WT in size (Fig. 2). The double mutant was tested on leaves only and produced symptoms similar to those of the *nuc1* single mutant.

**FIG 3:**
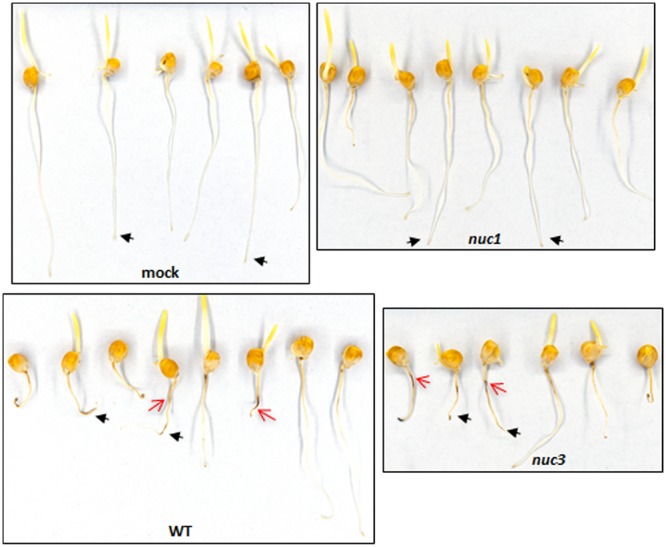
Virulence of the *nuc1* mutant is reduced on roots. Comparison of root necrosis phenotypes of mock-, WT-, the *nuc1* mutant-, and *nuc3* mutant-inoculated roots. The mock- and *nuc1* mutant-inoculated roots look similar, while the WT-inoculated and *nuc3* mutant-inoculated (plus *nuc2*, *nuc4*, and *nuc5* mutants, not shown) roots show necrosis (red arrows). Average length to root tip is indicated by black arrows.

Root symptoms (brown lesions) caused by the *nuc1* mutant were reduced compared to those caused by the WT or any of the other mutant strains (Fig. 3). Roots grew more and were much less brown than the short heavily diseased roots inoculated with WT (Fig. 3, red arrows). Mock-inoculated roots were the longest and did not show browning.

Thus, strains lacking *NUC1* are reduced in virulence on both above- and below-ground plant tissues.

### The reduced virulence phenotype can be rescued by the addition of DNase I.

**FIG 4:**
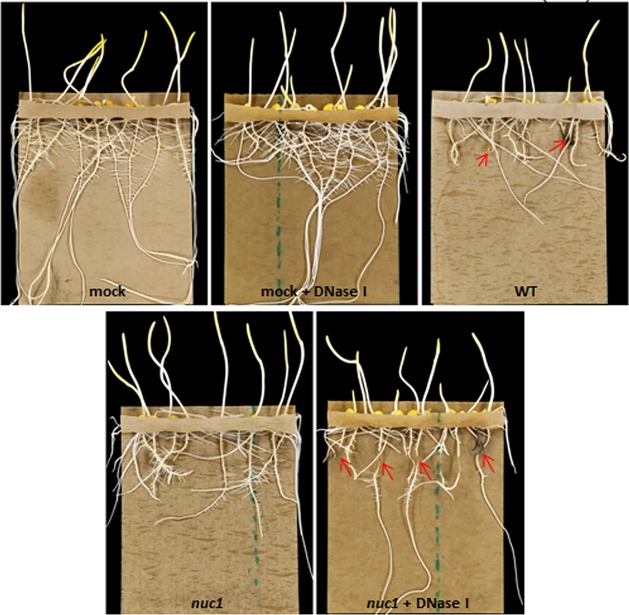
The *nuc1* mutant is rescued by the addition of DNase I. Roots treated with the mutant plus DNase I show necrotic symptoms (red arrows), while those without DNase I do not. The mock control was water instead of spores; the addition of DNase I to the control had no deleterious effect on the roots.

When DNase I was added to the conidial suspension used in pouch inoculations, symptoms (brown lesions) caused by the *nuc1* mutant (Fig. 4, bottom right, red arrows) were more similar to those caused by the WT (Fig. 4, top right) than those caused by the *nuc1* mutant without DNase treatment (Fig. 4, bottom left). This suggests that the *nuc1* mutant can be rescued by exogenous addition of pure enzyme. Application of DNase alone did not affect the roots (Fig. 4, compare mock, top left, with mock + DNase, top middle).

Our findings imply that the otherwise infection-competent *nuc1* mutant is debilitated and that the Nuc1 DNase produced by the WT is a virulence factor.

### The Nuc1 protein is a candidate secreted DNase.

**FIG 5:**
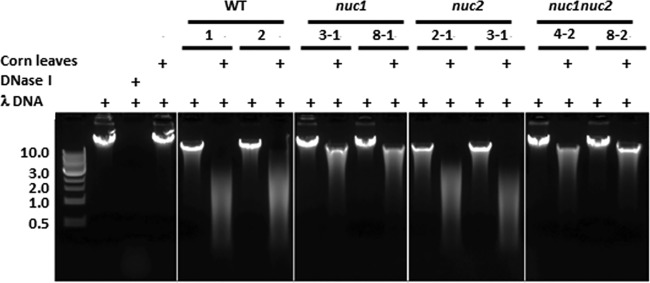
*C. heterostrophus* secretes DNases, and secretion is induced by plant tissue. WT, *nuc1* mutant, *nuc2* mutant, and *nuc1 nuc2* double-mutant filtrates degrade intact λ DNA in the presence of plant material. The *nuc1* single mutant and *nuc1 nuc2* double mutant degrade lambda DNA less well than the WT or the *nuc2* single mutant. This indicates that DNase(s) are secreted by the fungus, that the *nuc1* mutant secretes a DNase that is important in DNA degradation, and that secretion is induced by host material. Left, size markers in kilobases. +, addition of corn leaf (CL) fragments, lambda DNA, or purified RQ1 RNase-free DNase. Culture filtrates examined were from WT strain C4, *nuc1* mutant strains 144206-3-1 and 8-1, *nuc2* mutant strains 149183-2-1 and 3-1, and *nuc1 nuc2* double-mutant strains 144206/149183-4-1 and 8-1. The negative-control reaction with λ DNA did not degrade the DNA, while the positive-control reaction with λ DNA plus DNase did. Also note that λ DNA was not degraded by the CL material.

Culture filtrates from WT C4 and *nuc1* and *nuc2* single and double mutants were tested for secreted DNase activity. Interestingly, the addition of corn leaves to the culture medium induced the secretion and/or the expression and secretion of one or more fungal extracellular DNases, as evidenced by degradation of lambda (λ) DNA (Fig. 5). Without the addition of corn leaves, λ DNA remained intact. Corn leaves alone did not degrade λ DNA. Filtrates from two independent strains (3-1 and 8-1) of the *nuc1* mutant showed a dramatic decrease in ability to degrade λ DNA compared to the WT filtrate. The *nuc2* mutant filtrates did not alter the DNase activity and degraded λ DNA as well as the WT. The *nuc1 nuc2* double-deletion mutant filtrates showed levels of DNase activity similar to the *nuc1* single-mutant filtrates. Minor residual DNase activity is evident in *nuc1* single- and double-mutant filtrates, indicating that additional unidentified secreted DNases were present.

These results indicate that the activity and secretion of Nuc1 are major contributors to overall DNase activity in WT culture filtrates and that they are induced by the presence of corn leaves.

### Nuc1 has DNase activity.

To confirm that the Nuc1 protein has DNase activity, portions of Nuc1 and Nuc2 were cloned (Fig. S3A and B), expressed in Escherichia coli, and purified on an amylose-resin column. Single purified maltose binding protein (MBP)-Nuc1 and -Nuc2 fusion protein bands are shown in Fig. S4, lanes E1 and E2. When the elution fractions containing the purified partial proteins were used to assay DNase activity, they both degraded the λ DNA in a dosage-dependent manner (Fig. S5A). In addition, this DNase activity is Mg^2+^ dependent (Fig. S5B).

10.1128/mBio.02805-18.3 FIG S3 Cloning of *NUC1* and *NUC2* for expression in E. coli. (A) Nuc1 (144206) as a NCBI BLASTp query number represents conserved consensus amino acids in the predicted active site of TatD type deoxyribonucleases. Red underline corresponds to the Nuc1 region cloned and assayed for cloned nuclease activity. (B) Nuc2 (149183) as a NCBI BLASTp query number represents conserved consensus amino acids in the predicted active site of TatD type deoxyribonucleases. Red underline corresponds to the Nuc2 region cloned and assayed for cloned nuclease activity. Download FIG S3, PDF file, 0.3 MB. Copyright © 2019 Park et al. 2019 Park et al. This content is distributed under the terms of the Creative Commons Attribution 4.0 International license.

10.1128/mBio.02805-18.4 FIG S4 Expression and purification of MBP-fusion proteins. Protein IDs 144206 (Nuc1) and 149183 (Nuc2) were induced for expression and purified on an amylose-resin column. Bands on the polyacrylamide gel indicate the purification process. Single elution fractions E1 and E2 indicate the purified proteins. (A) SDS-polyacrylamide gel of Nuc1MBP-fusion protein purification. The calculated MBP-Nuc1 protein size is 65.4 kDa. (B) SDS-polyacrylamide gel of Nuc2 MBP-fusion protein purification. The calculated MBP-Nuc2 protein size is 76.0 kDa. MBP, maltose binding protein; SM, protein size marker (catalog no. 10748-010, BenchMark prestained protein ladder; Invitrogen); L, loading; F, flowthrough; W1 to -3, washing; E1 to -4, elution fractions of amylose-resin column. Download FIG S4, PDF file, 0.3 MB. Copyright © 2019 Park et al. 2019 Park et al. This content is distributed under the terms of the Creative Commons Attribution 4.0 International license.

10.1128/mBio.02805-18.5 FIG S5 DNase assay of recombinant Nuc1 and Nuc2 proteins. (A) The purified MBP-fusion proteins (Fig. S4) were tested for DNase activity. Note that the column elution buffer (fifth lane from left) did not affect λ DNA degradation or RQ1 DNase activity. The last washing fractions from the column were tested and shown not to degrade λ DNA (W3, right two lanes), confirming that the RQ1 DNase was effective in degrading E. coli host genomic DNA released during cell lysis. The 144206 (0.84, 1.68, and 2.6 µg) and 149183 (0.28, 0.56, and 0.87 µg) purified proteins were used for the assay. DNase, RQ1 DNase (catalog no. M610A; Promega); Elu buffer, elution buffer fraction; MBP-144206 and MBP-149183 W3, third washing fraction of column. (B) The purified proteins were assayed for Mg^2+^ dependency. MgSO_4_ concentrations of 0, 5, 10, 25, and 50 mM were tested. The 144206 fusion protein (1.68 µg) and 0.56 µg of 149183 fusion protein were used for the assay and incubated for 1 h and 2 h. Samples without added Mg^2+^ did not show activity. DNA digestion activity of the purified proteins deceased as the concentration of Mg^2+^ increased beyond 10 mM. DNase, RQ1 DNase. Download FIG S5, PDF file, 0.5 MB. Copyright © 2019 Park et al. 2019 Park et al. This content is distributed under the terms of the Creative Commons Attribution 4.0 International license.

These findings suggest that the cloned portions of the Nuc1 and Nuc2 proteins have DNase activity and, together with the culture filtrate assays, are Mg^2+^-dependent extracellular DNases.

In addition, we made a construct to produce a C-terminal HA-tagged version of Nuc1 and used it to complement the *nuc1* mutant strain 4-1 (Fig. S6). Two independent complemented strains (7-1 and 9-1) were returned to WT-level growth and also restored virulence to corn to WT levels (Fig. S7). This indicated that the hemagglutinin (HA)-tagged gene has WT-level function in terms of growth and virulence. However, when culture filtrates of WT and the HA-tagged complemented strain were assayed by Western blotting, we failed to detect the protein. In troubleshooting this, we first asked if the HA-tagged version of the gene was expressed and verified expression by reverse transcription-PCR (RT-PCR) using three different primer pairs (Fig. S8A). Primer set WW105/HJ3 produced the same-sized bands with all samples (Fig. S8B), while primer set WW105/WW106 did not produce bands, as expected (not shown), since primer WW106 spans an intron junction (Fig. S8A). The bands produced using the HA-fusion strain templates and primer pair WW105/HJ34 were much weaker (Fig. S8B) than the bands produced using the same templates with the WW105/WW106, where the intensity was similar to that of the WT band. There were no bands using WT RNA as the template, as expected. The low level of expression may account for our failure to detect the Nuc1-3HA-tagged protein by Western blotting and will be the subject of future investigation.

10.1128/mBio.02805-18.6 FIG S6 Strategy for complementation of strain 144206 4-1 with HA-tagged 144206. (A) As described in Materials and Methods. Mutant strain 144206 4-1 (*nuc1,* HygR) was transformed with a PCR product carrying the 5′ flank (F1-1), *NUC1*-3HA, and 3′ flank (F2-1) PCR products combined into one using WW99/WW102 primers and a PCR product carrying the 5′ flank (F2-2)-*nptII*-3′ flank (F3) PCR products combined into one using WW271/WW272 (Table S1). (B) The *NUC1*-3HA gene insertion at the native locus was confirmed by diagnostic PCR using primer pairs: UF (WW103)/HJ34 (product 2) for *NUC1*-3HA insertion, FP (WW105)/RP(WW106) for the *NUC1* gene only (product 1), and Ttrpc(BH14)/DR3(WW273) (product 3) for *nptII* insertion. Note that WT has the *NUC1* gene only, the complemented strain 144206-4-1 lacks both products 1 and 2 but has product 4, and all four candidate strains have products 1, 2, and 3, but not 4. Download FIG S6, PDF file, 0.4 MB. Copyright © 2019 Park et al. 2019 Park et al. This content is distributed under the terms of the Creative Commons Attribution 4.0 International license.

10.1128/mBio.02805-18.7 FIG S7 Complementation with *NUC1*-3HA restores WT growth and virulence to corn. (A) WT, the *nuc1* mutant strain 4-1, and two strains (7-1 and 9-1) of the *nuc1* mutant complemented with *NUC1*-3HA. Note that the latter grow as well as WT. Strains were grown on CMX. (B) The same strains were spray inoculated on corn and 3 days after inoculation. The complemented strains are similar to the WT in virulence to corn. Download FIG S7, PDF file, 1.0 MB. Copyright © 2019 Park et al. 2019 Park et al. This content is distributed under the terms of the Creative Commons Attribution 4.0 International license.

10.1128/mBio.02805-18.8 FIG S8 Expression of HA-tagged *NUC1.* (A) Topology of *NUC1* with an HA tag at the 3′ end. Introns are indicated in yellow and the HA tag is indicated in red. Total RNA was extracted from the two complemented *NUC1-*3HA strains (7-1 and 9-1) plus the WT and treated with DNase, and expression of the gene verified by RT-PCR using different primer pairs (Table S1). (B) Primers to the actin gene (Table S1) were used as a control. Primer set WW105/HJ3 produced the same sized bands with all samples. The bands produced using the HA-fusion strain templates and primer pair WW105/HJ34 were much weaker than the bands produced using the same templates with the WW105/HJ3 pair, where the intensity was similar with WT band. The low level of expression may account for our failure to detect the *NUC1*-3HA-tagged protein by Western blotting. Download FIG S8, PDF file, 0.4 MB. Copyright © 2019 Park et al. 2019 Park et al. This content is distributed under the terms of the Creative Commons Attribution 4.0 International license.

### Double mutants lacking *NUC1* and the ability to produce an extracellular matrix are much reduced in virulence to maize.

To determine the phenotype of a double mutant lacking *NUC1* and an extracellular matrix (ECM) around spores and hyphae, the *nuc1* mutant (*hygR MAT1-2 ECM1 nuc1*; strain 144206-4-1) was first crossed to strain CB7 (*MAT1-1 ECM1 NUC1*) to obtain an opposite mating type progeny (*MAT1-1 ECM1 nuc1*). Then, one of these progeny (strain #36) was crossed to untagged *ecm1* mutant BC3-58 (*MAT1-2 ecm1 NUC1*). Progeny were collected and screened first for resistance to hygromycin B, which marks the deletion of *NUC1*. HygR progeny were then screened for presence of an extracellular matrix by India ink staining, selecting those that had no extracellular matrix.

Double mutants were more reduced in virulence ability than the *nuc1* and *ecm1* single mutants, both of which were reduced compared to the WT; *nuc1* mutant lesions were smaller than those of the *ecm1* mutants (Fig. S9). Note that both single mutants are at WT levels with respect to conidiation, germination, and initial penetration events on maize leaves. We hypothesize that the fungal extracellular matrix may be important for the delivery and protection of virulence factors such as the Nuc1 exDNase. The order of virulence ability we observed from greatest to least, WT*, ecm1, nuc1,* and *ecm1 nuc1,* fits with the notion that deletion of *NUC1* critically impacts ability to digest plant-secreted DNA and thus, infection. The deletion of *ECM1,* however, eliminates the outer extracellular matrix (carbohydrates, proteins, and presumably DNA) but leaves the extracellular inner protein layer of the fungus intact (31); thus, some Nuc1 can still be delivered but less efficiently.

10.1128/mBio.02805-18.9 FIG S9 Phenotypes of *nuc1 ecm1* double mutants. (A) Virulence of WT, *nuc1* mutant, *ecm1* mutant, and the *nuc1 ecm1* double mutant on W64-N maize. Note that the double mutant has smaller lesions than any other strain. Inset, lesion measurements and statistical significance. (B) The *ecm1* mutant and the *nuc1 ecm1* double mutant lack an extracellular matrix (black arrows). Download FIG S9, PDF file, 1.0 MB. Copyright © 2019 Park et al. 2019 Park et al. This content is distributed under the terms of the Creative Commons Attribution 4.0 International license.

### Nuc1 and Nuc2 proteins are widely conserved in filamentous fungi.

Nuc1 from strain C4 is encoded on JGI scaffold_19:406934-408049 and is annotated as a TatD-related Mg^2+^-dependent DNase with no predicted transmembrane domains. As a query against JGI protein catalogs, the full Nuc1 protein is highly conserved in Dothideomycetes, Eurotiomycetes, and Lecanoromycetes, but only portions of the protein are conserved in most Leotiomycetes and Sordariomycetes. Whether or not orthologs of *NUC1* function similarly in other pathogenic fungi or in saprobes remains to be tested.

### Corn roots secrete extracellular DNA.

**FIG 6:**
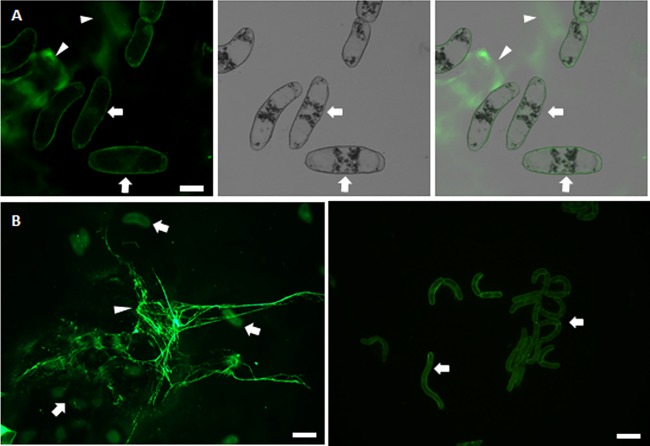
Corn root cap cells secrete DNA that is degraded by DNase I. (A) Corn roots were touched to glass slides, causing border cells (arrows) to slough off. Slides were stained with Sytox green, which stains extracellular DNA (arrowheads) or DNA in dead cells. Left, fluorescence; middle, differential interference contrast (DIC); right, merged. Scale bar is 20 μm. (B) DNase treatment degrades extracellular DNA. Left, corn roots and root cap border cells (arrowheads), Sytox green staining outside border cells (arrows); right, no exDNA staining after DNase treatment. Scale bar is 50 μm.

It has been documented previously that extracellular DNA is associated with corn roots (16, 32). We confirmed this finding by examining material stained with Sytox green left on glass slides after touching corn roots to the slide. Figure 6A shows that Sytox green fluorescence can be found outside detached root cap cells (yellow arrowheads). In addition, Sytox green-stained materials were absent or highly reduced when DNase was included in the staining solution (Fig. 6B).

## DISCUSSION

A DNase produced by the maize pathogen C. heterostrophus has been demonstrated to be involved in virulence of the fungus to its plant host by comparison of the original WT strain and an isogenic mutant made by deletion of the gene encoding the candidate DNase. A significant reduction in average lesion size on leaves or in browning of roots was apparent when the WT and mutant were compared. Complementation of the mutant with a WT copy of *NUC1* restores WT-level virulence. Furthermore, the reduced virulence phenotype can be rescued by the addition of DNase I. Virulence assays were conducted both on leaves (Fig. 2), the best documented site of C. heterostrophus infection (33, 34), and on roots (Fig. 3 and 4), which have not been reported previously as infection portals.

That the DNase is likely secreted was demonstrated by the fact that WT and *nuc2* culture filtrates, assayed after 3 days of fungal growth in liquid medium, had degraded λ DNA within 10 min. The *nuc1* single- and *nuc1 nuc2* double-mutant culture filtrates showed less degradation, supporting the hypothesis that Nuc1 is associated with most of the degradation activity. Furthermore, in all cases, degradation of DNA was dramatically induced by the presence of pieces of corn leaf material in the medium used to grow the fungus. We note that there was a very small amount of DNA degradation in the filtrates from WT and the *nuc2* mutant (both of which carry *NUC1*) when corn leaves were not included in the culture medium, which indicates that the host material is not absolutely required for activity. Corn leaves alone did not degrade λ DNA. There are 30 candidate DNase-encoding genes in the C. heterostrophus genome, but only a fraction of these are predicted to be secreted. We chose five of the latter for deletion and testing of mutants for altered virulence and found that only one (*NUC1*) of the five genes had a reduced virulence phenotype compared to the WT. This suggests that the circumstance under which the Nuc1 functions is highly specific.

To provide evidence that Nuc1 has DNase activity and is the enzyme responsible for DNA degradation, we cloned and expressed a portion of the gene encoding the predicted DNase domain in E. coli and then assessed the ability of the purified protein to degrade DNA, which it did. We note that alignments of the Nuc1 protein to proteins annotated as TatD deoxyribonucleases (NCBI, conserved domains) show that three of the five TatD annotated active-site residues are conserved in Nuc1 and Nuc2 (Fig. S3A and B). Our Nuc1 and Nuc2 recombinant proteins encompass two or three of these, respectively; nevertheless, both have DNA-degrading activity. Future experiments will focus on identification of amino acid residues important for DNase activity, including point mutations affecting the canonical residues. To date, our experiments demonstrate that Nuc1 and Nuc2 activity is Mg^2+^ dependent, as are the first described virulence-associated extracellular DNases in the animal bacterial pathogens Streptococcus and Staphylococcus spp. (3, 10, 11) and the NucA and NucB DNases from the plant bacterial pathogen R. solanacearum (19).

There are no previous reports of deletion of a fungal exDNase-encoding gene from either an animal or plant pathogen coupled with concomitant assay of the mutant for alterations in virulence to the host compared to the WT strain. We speculate that Nuc1 functions to degrade plant-secreted DNA that is a component of a complex matrix secreted by plant cells, akin to neutrophil extracellular traps of animals. We also acknowledge that although we have identified a fungal extracellular DNase that is important for virulence to the host maize, in this report, we have not demonstrated what happens when this DNA interacts with WT and *nuc1* strains. *In planta* demonstration of plant-secreted extracellular DNA is technically challenging and complicated by the fact that C. heterostrophus also secretes a complex extracellular matrix (Fig. S9) that may contain DNA, as has been shown for Aspergillus fumigatus (35). Our *nuc1* and *ecm1* single and *nuc1 ecm1* double mutants (Fig. S9) may assist in resolving host-versus-pathogen contribution in future experiments. Given that *NUC1* is conserved both in pathogens and saprobes, and that secretion of DNA by organisms, including hosts and their microbes, is apparently commonplace, we speculate that the exDNA/exDNase mechanism may be broadly involved in host interactions with microbes of diverse lifestyles, e.g., pathogens, endophytes, symbionts, biocontrol agents, etc.

These data provide support for a common exDNA/exDNase defense/counter defense virulence mechanism used by animals, plants, and their fungal and bacterial pathogens.

Components of the mechanism could be novel targets for the control of plant disease.

## MATERIALS AND METHODS

### Fungal strains and plant materials.

C. heterostrophus strain C4 (*Tox1*^+^ *MAT1-2* ATCC strain 48331), all strains derived from it, strain CB7 (B30-A3-R-20) (36), and strain *ecm1* (31) were grown on complete medium with xylose (CMX) under a 16-h light/8-h dark regimen at 23°C, as previously described (34, 37).

Corn cultivar W64A-N was used to assay virulence. All plants were grown in a growth chamber with a light cycle of 16 h light/8 h dark at 24°C.

### Identification of DNase-encoding genes.

The C. heterostrophus strain C4 genome was searched for proteins annotated as DNases (http://genome.jgi.doe.gov/CocheC4_1/CocheC4_1.home.html). In addition, BLAST searches (38) with previously identified fungal DNases (e.g., SCN1 [GI:633129] from Schizosaccharomyces pombe and Fusarium solani f. sp. *phaseoli* [GenBank accession no. AAD53090.1 GI:5823280]) as queries were conducted. To identify candidate secreted DNases, each candidate protein was screened for secretion signals using SignalP (30) and SecretomeP (29), which generate nonclassical neural network (NN) secretion scores.

### Deletion of DNase-encoding genes.

A subset of genes encoding proteins (JGI protein IDs 144206 [Nuc1], 149183 [Nuc2], 33717 [Nuc3], 122478 [Nuc4], and 83474 [Nuc5]) with NN secretion scores of approximately 0.5 or higher were chosen for deletion in strain C4, following a PCR-based split-marker homologous recombination technique (33, 34), except that the selectable marker was amplified as a single fragment (Fig. S1A). Note that not all of these proteins have a secretion signal as determined by SignalP. Transformants were selected for resistance to hygromycin B, conferred by the *hygB* gene, and screened by PCR for absence of the DNase*-*encoding gene(s) and targeted insertion of the selectable marker into the native locus of each gene using previously described diagnostic PCR protocols (Fig. S1B and C) (39, 40). Primers used for gene deletion and for verification of gene deletion are listed in Table S1.

10.1128/mBio.02805-18.10 TABLE S1 Primers used for gene deletion and verification of insertion at target site. Download Table S1, PDF file, 0.01 MB. Copyright © 2019 Park et al. 2019 Park et al. This content is distributed under the terms of the Creative Commons Attribution 4.0 International license.

To generate a double mutant lacking genes encoding both Nuc1 and Nuc2 proteins, the *NUC2* gene encoding 149183 was deleted in one of the *nuc1* single mutants (strain 144206-4-1, hygromycin B resistant [*hygB*^r^]) using the strategy described above but with the *nptII* gene (41) for resistance to G418 (catalog no. 61-234-RG; Corning Cellgro) as the selectable marker. Double mutants were selected for resistance to both hygromycin B and G418 (42). Deletion of the *NUC2* gene was confirmed by PCR (Fig. S1D). At least two independent mutants for each gene deleted or for the double mutant were purified by single conidiation to eliminate heterokaryons.

### Complementation of the *nuc1* mutant.

Complementation of the *nuc1* mutant (strain 144206-2-1) was based on protocols described by Wang et al. (40). Briefly, the *NUC1* open reading frame (ORF) plus 5′ and 3′ flanking sequences were amplified from WT (Fig. S2A). The *nptII* cassette from pII99 (41) flanked at the 5′ end by the *NUC1* 3′ flanking sequence and at the 3′ end by a sequence immediately downstream of the *NUC1* 3′ flanking sequence, generated by overlapping PCR, was used for selection. The *NUC1* ORF plus flanking sequences and the *nptII* cassette were cotransformed into the *nuc1* mutant. Transformants were selected for resistance to G418 and sensitivity to hygromycin B, purified by single conidiation, and screened with pairs of PCR primers (Table S1 and Fig. S2B) for confirmation of integration of the construct. For this, a set of primers (WW105/WW106) internal to *NUC1* and two sets of primer pairs (PtrpC/WW269 and TtrpC/WW273), in which one primer was internal to the introduced selectable marker and the other was external to either the 5′ or 3′ flanking region used to introduce the *NUC1* gene, were used (Fig. S2B).

### Assays for growth, conidiation, conidial germination, and appressorium formation.

WT and mutant strains were grown in triplicate as described above and growth characteristics observed visually. Conidia were harvested from mature plates at ∼1 week, counted with a hemocytometer, and assayed for germination rate and ability to form appressoria on glass slides. For the latter, a sterile needle was used to scrape and capture conidia from colony surfaces, then conidia were placed in a drop of water on a glass slide housed in a humid chamber. Germination and appressorium formation were tracked for about 6 h. Photographs were taken using a Nikon E600 microscope with differential interference contrast optics and a Spot 14.2 digital camera.

### Virulence of C. heterostrophus mutants and wild type on maize.

Two different methods, leaf spray and root inoculation, were used to test the virulence of mutant and wild-type strains on Zea mays cv. W64A-N.

For leaf spray inoculation, strains were grown for 10 days, conidia harvested, and sprayed on leaves of 3-week-old plants (∼2 ml, 10^3^ conidia/ml) according to previously described protocols (34, 41).

For each fungal strain, at least four replicates (i.e., inoculation of four independent plants) were used, and experiments were repeated three times. Photographed leaves were imaged in Photoshop CS5, and the length of necrotic lesions was measured with a ruler. Statistical analysis was done by a *t*-test.

For root inoculation (43, 44), W64A-N corn seeds were surface sterilized with 5% bleach for 7 min then rinsed with sterilized water 7 to 10 times. After soaking in sterilized water for 2 h, imbibed seeds were spread on sterilized filter paper overlaid on 1% water agar and incubated in the dark for 4 days. Cellophane growth pouches (Mega International) were used for further growth. Seedlings with radicles of ∼25 mm that had full sets of border cells (45) were inoculated by application of a 50-μl conidial suspension (10^5^/ml) and then placed in pouches containing 16 ml of sterilized water. Pouches were placed in the dark at 23°C for 1 week then photographed. Water, instead of conidial suspensions, was used as mock control.

### Treatment with DNase I.

For nuclease treatments, 1.2 units DNase I (Promega) was added to the 50-µl conidial suspension (∼10^5^ conidia/ml) immediately before pouch inoculation. Root tips treated with DNase I in water without fungal spores served as controls (17).

### Activity assays of native and purified DNase.

To test for secreted DNase activity of *nuc1* or *nuc2* single and *nuc1 nuc2* double mutants and the WT, strains were first grown for 1 week on CMX (42). Conidia were harvested by applying minimal medium (37) with xylose (MMX) to mycelial surfaces and rubbing with a sterilized rubber policeman. Conidia were resuspended in MMX at a concentration of 1 × 10^5^ conidia/ml. For the assay, 400-µl (4 × 10^4^ conidia) aliquots of each sample were added to 2-ml Eppendorf tubes with or without six pieces (4 by 4 mm) of 2- to 3-week-old third leaves of maize (W64A-N). Leaves were first sterilized with 5% bleach solution for 7 min and washed with sterilized H_2_O 7 times. The cultures were incubated for 3 days at room temperature (25°C) with gentle shaking (25 rpm). Tubes were centrifuged at 11,000 × *g* for 5 min and supernatants used for the DNase activity assay. The assay reaction mixture containing 2.5 µg of λ DNA (catalog no. N3011S; New England BioLabs, Inc.) and 5 µl of culture supernatant was incubated for 10 min at 37°C. Reactions were run on 1% agarose gels at 100 V for 15 min. As a positive control for λ DNA degradation, 1 unit of RQ1 DNase (catalog no. M610A; Promega) was used.

To test DNase activity of recombinant Nuc1 and Nuc2 proteins (purification described below), reaction mixtures contained 2.5 µg of λ DNA and 0.84 to 2.6 µg of the purified Nuc1 or 0.28 to 0.87 µg of Nuc2 MBP-fusion proteins. The mixture was incubated at 37°C for 2 h and then run on 1% agarose gels.

### Assay for Mg^2+^ dependency.

Tris-HCl (40 mM; pH 8.0) reaction buffer was used for the assay. λ DNA (2.5 µg) and 1.68 µg of purified Nuc1 or 0.58 µg of Nuc2 proteins were added to the assay mixture with or without MgSO_4_ (5, 10, 25, or 50 mM). The conditions and detection of the DNA degradation were as described above for the activity assay.

### DNase expression plasmid construction and enzyme purification.

To verify that Nuc1 and Nuc2 proteins had DNase activity, the proteins were expressed, purified, and assayed for activity. The coding sequences of protein IDs 144206 and 149183 were obtained from the JGI website (http://genome.jgi.doe.gov/CocheC4_1/CocheC4_1.home.html). Five hundred twenty-eight base pairs of the DNA sequence corresponding to protein ID 144206 (scaffold_19: 407843 to 407306) and 807 bp of the DNA sequence corresponding to protein ID 149183 (scaffold_25: 86457 to 87263) that included the candidate DNase domains were amplified with primers HJ10/11 (144206) and HJ8/12 (149183) using Phusion DNA polymerase (catalog no. M0530S; NEB) (Table S1). PCR products were cloned into the pCR-Blunt vector using the Invitrogen Zero Blunt PCR cloning kit. The cloned DNAs were sequenced (Biotechnology Service Center, Cornell University Institute of Biotechnology). Plasmids with cloned inserts were digested with EcoRI, and fragments were cloned into the pETMAL expression vector. Sequences and their directions were confirmed by sequencing, as described above. The expression vectors were transformed into the expression host [E. coli BL21(DE3)].

To express and purify proteins, bacteria containing expression plasmids were inoculated into 2 ml lysogeny broth (Luria broth [LB]) medium containing 50 µg kanamycin/ml and cultured overnight at 37°C at 200 rpm in an incubator with shaking. Five hundred microliters of the cultures was inoculated into 50 ml LB with kanamycin and incubated for 3 h under the same conditions. To induce protein expression, isopropyl-β-d-thiogalactopyranoside (IPTG) solution was added to 0.1 mM concentration and the mixture incubated at 18°C for 2 h at 200 rpm. The cells were harvested by centrifugation at 4,000 × *g* and 4°C for 20 min, and the cell pellets were resuspended in 2.5 ml of column buffer (20 mM Tris-HCl [pH 7.4], 0.2 mM NaCl, and 1 mM EDTA). Lysozyme (final concentration, 0.1 mg/ml; catalog no. L-6878; Sigma) and RQ1 DNase (final concentration, 2 U/ml, catalog no. M610A; Promega) were added and the mixtures incubated at 37°C for 30 min for cell lysis. The cell lysates were centrifuged at 12,000 × *g* for 20 min at 4°C, and the supernatant (cell extract) was loaded on a column containing amylose-resin (catalog no. E8021S; NEB), which was preequilibrated 5 times with column buffer. The column was washed 3 times with column buffer and 3 times with elution buffer (20 mM Tris-HCl [pH 7.4], 0.2 mM NaCl) without maltose. The bound proteins were eluted with elution buffer (20 mM Tris-HCl [pH 7.4], 0.2 mM NaCl, and 10 mM maltose). All column operations were performed at 4°C. The purified protein concentration was measured by a Bradford assay (46), and the protein bands were confirmed by electrophoresis on a 10% SDS-polyacrylamide gel.

### Constructing an HA-tagged version of protein 144206.

The open reading frame of Nuc1 without the stop codon was amplified from WT DNA with primer pair HJ27/HJ28 (Table S1) by PCR. The purified PCR product was cloned into the pDONR 221 vector, and then the Nuc1-no stop sequence was cloned to pGWB414 to make a Nuc1-3HA fusion using the Gateway cloning method (47). The sequence and correct reading frame were confirmed by sequencing.

To generate a strain carrying the HA-tagged version of the Nuc1 protein, mutant strain 144206 4-1 (HygR) was transformed with 5′ flank-144206-3HA-3′ flank and 5′ flank-*nptII*-3′ flank DNA fragments. 5′ flank (F1-1), *NUC1*-3HA, and 3′ flank (F2-1) PCR products were produced using primer pairs WW99/HJ30, HJ33/HJ34, and HJ35/WW270. 5′ flank, *nptII,* and 3′ flank PCR products were produced using primer pairs WW269/WW270 (F2-2), m13F/m13R (*nptII*), and WW271/WW272 (F3) (Table S1). The three PCR products were first combined into one fragment using primers WW99/WW102 for the F1-1-144206-3HA-F2-1 fragment and WW269/WW272 for the F2-2-*nptII*-F3 fragment. Transformants were selected on G418 as described by Turgeon et al. (42). G418-resistant transformants were screened for loss of HygR. The *NUC1*-3HA gene insertion at the native locus was confirmed by diagnostic PCR using primer pairs UF (WW103)/HJ34 for *NUC1*-3HA insertion into the native *NUC1* 5′ flank, FP (WW105)/RP(WW106) for the *NUC1* gene only, and Ttrpc/DR3(WW273) for *nptII* insertion into the native *NUC1* 3′ flank.

Strains *NUC1*-3HA 7-1 and 9-1 were cultured as described above for the DNase degradation assay. Supernatants were filtered using sterilized Whatman filter paper no. 1 (catalog no. 1001-110) and concentrated 40 to 85 times using Amicon Ultra 10-K centrifugal filter devices. Whole protein was extracted, separated on a 12% SDS-acrylamide gel, and transferred to nitrocellulose membrane (catalog no. 162-0145; Bio-Rad). The HA-fusion protein was blotted with HA antibodies (catalog no. PAI-985; Invitrogen; or catalog no. 51064-2-AP; Proteintech).

### Crosses to create a *nuc1 ecm1* mutant.

To obtain a double mutant lacking the *NUC1* gene and an extracellular matrix, the *nuc1* mutant (*hygR MAT1-2 ECM1 nuc1*; strain 144206-4-1) was first crossed to strain CB7 to obtain a *MAT1-1 ECM1 nuc1* progeny. Mating type was confirmed by PCR with diagnostic *MAT1-1-1* primers (MAT1-1-1-L1 and MAT1-1-1-R1; Table S1). Progeny #36 was crossed to untagged *ecm1* mutant BC3-58 (B220a.P1.4.3 [31]) generated in the lab of Charlotte Bronson, Iowa State (*MAT1-2 ecm1 NUC1*). Progeny were collected and screened first for resistance to hygromycin B. HygR progeny were then screened for presence of an extracellular matrix by India ink staining, selecting those that had no extracellular matrix and thus were *hygR ecm1*.

### Assay for extracellular corn root DNA.

Corn seeds were sterilized with 5% bleach solution, rinsed 7 times with sterilized water, and then allowed to imbibe sterilized water for 2 h. Imbibed seeds were placed on sterilized filter paper on top of 1% water agar in petri dishes and then incubated in the dark for 2 to 3 days.

To detect DNA secretion from roots, seedlings were placed on glass slides and root tips treated with 10 to 20 µl of staining solution (100 µl of sodium acetate buffer [pH 5.5] containing 1 µl of Sytox green nucleic acid stain dye [5 mM solution in dimethyl sulfoxide {DMSO}, catalog no. S7020; Invitrogen]) with or without RQ1 DNase (catalog no. M6101; Promega). After 10 to 20 min, roots were carefully removed, and the remaining solution was examined under a fluorescence microscope (Leica DM5500) or SP5 Leica confocal microscope.
