# Supplementary material for: A DNase from a Fungal Phytopathogen Is a Virulence Factor Likely Deployed as Counter Defense against Host-Secreted Extracellular DNA
Source: mBio. 2019 Mar 5;10(2):e02805-18. doi: 10.1128/mBio.02805-18 (PMC6401486; doi:10.1128/mBio.02805-18)
Supplement: FIG S1 [file mBio.02805-18-sf001.pdf]

A. Strategy to delete DNase-encoding genes

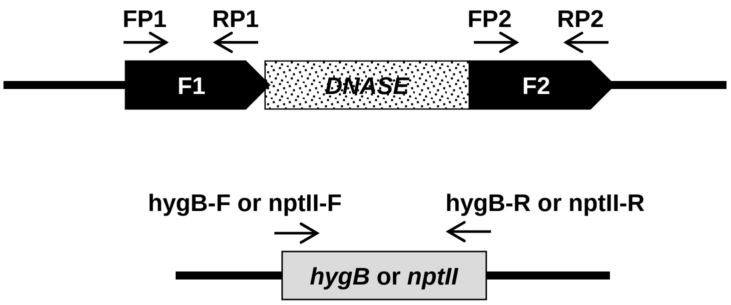

B. Proof of deletion of DNase-encoding genes

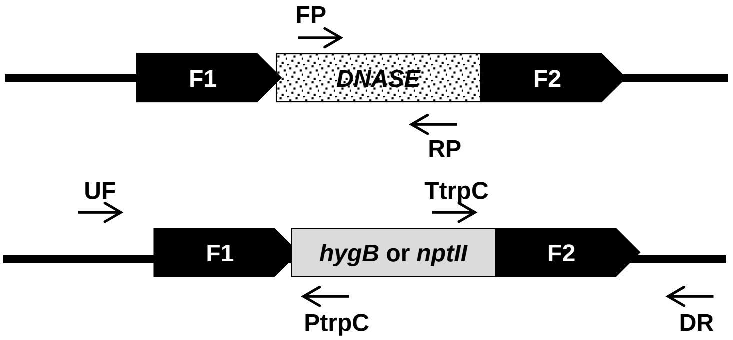

C. PCR evidence of deletion of DNase-encoding genes

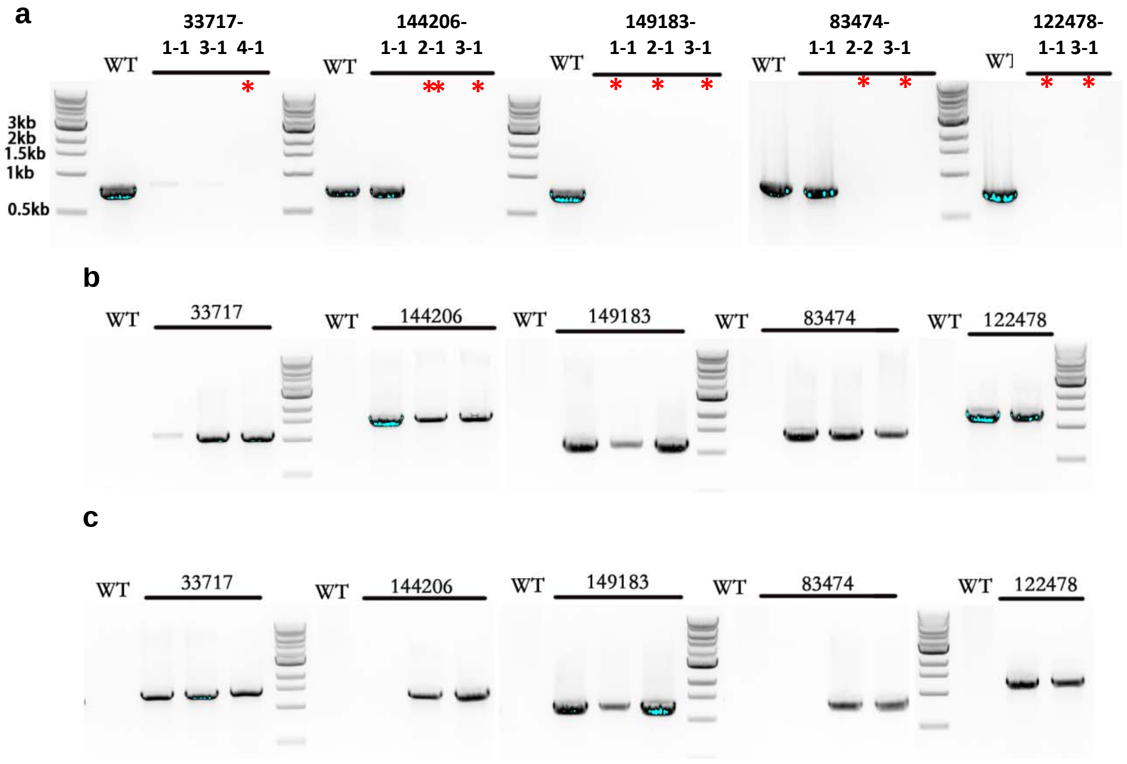

Figure S1A-C

D. PCR confirmation of *nuc1nuc2* double mutants

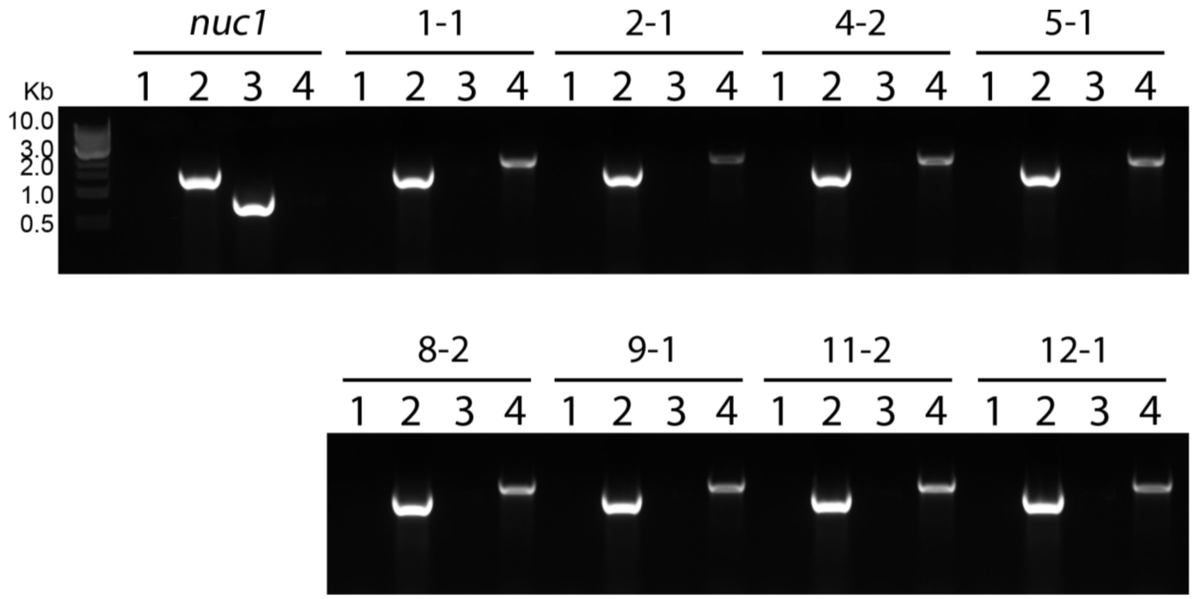

Figure S1D
