# Supplementary material for: A DNase from a Fungal Phytopathogen Is a Virulence Factor Likely Deployed as Counter Defense against Host-Secreted Extracellular DNA
Source: mBio. 2019 Mar 5;10(2):e02805-18. doi: 10.1128/mBio.02805-18 (PMC6401486; doi:10.1128/mBio.02805-18)
Supplement: FIG S2 [file mBio.02805-18-sf002.pdf]

A. Strategy to complement *nuc1* (144206) mutant with WT *NUC1* (144206)

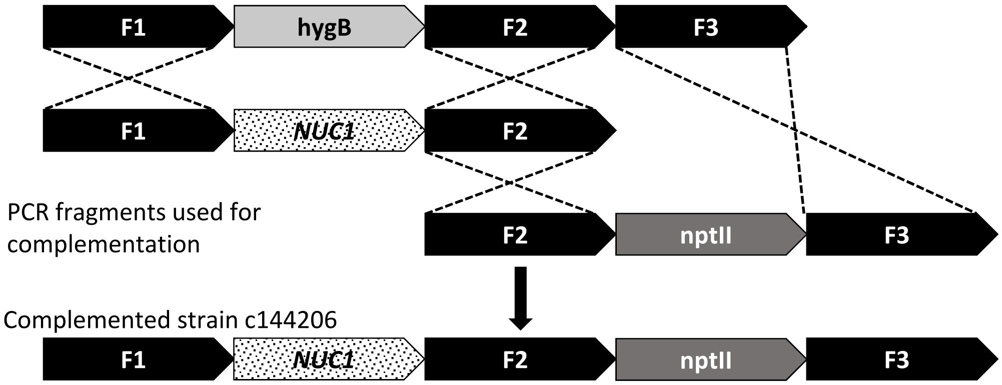

B. Complementation of *nuc1* (144206) mutant with WT *NUC1*

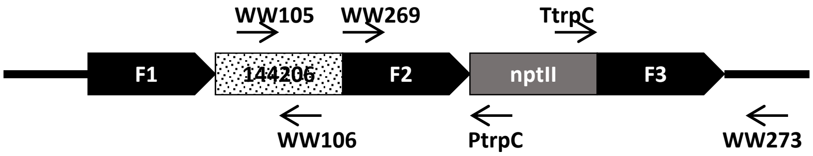

C.

		WT	*nuc1*	c144206
a	WW105/WW106	0.8kb	-	0.8kb
b	PtrpC/WW269	-	-	1.7kb
c	TtrpC/WW273	-	2.3kb	1.1kb

D.

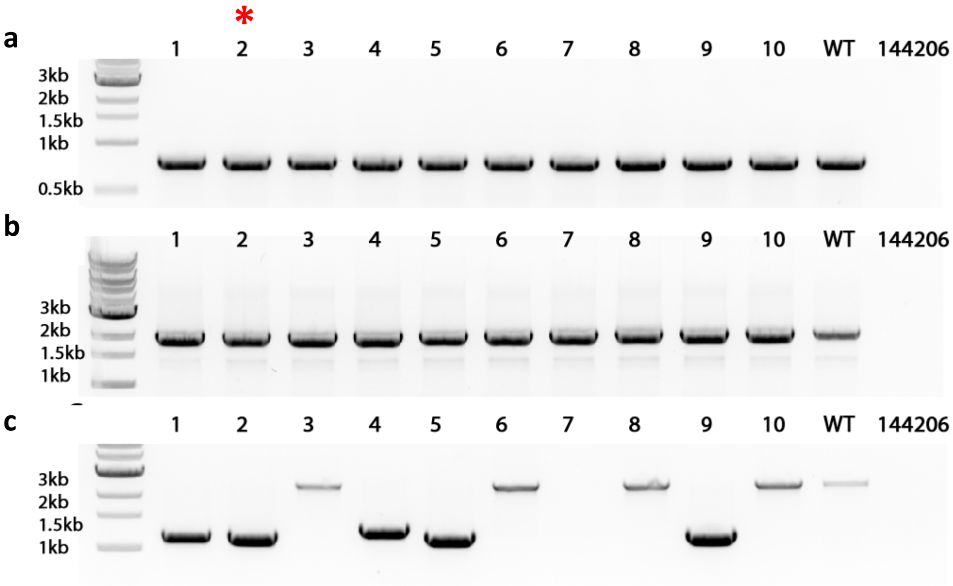

Figure S2
