# Supplementary material for: A DNase from a Fungal Phytopathogen Is a Virulence Factor Likely Deployed as Counter Defense against Host-Secreted Extracellular DNA
Source: mBio. 2019 Mar 5;10(2):e02805-18. doi: 10.1128/mBio.02805-18 (PMC6401486; doi:10.1128/mBio.02805-18)
Supplement: FIG S3 [file mBio.02805-18-sf003.pdf]

JGI 144206 (Nuc1) = query

```
Feature 1                  #  #
1J6O_A        13 XVDTHAHLH.[10].VISSFEEN.[2].EFVVNVGV.[5].KKSLDL.[7].FCSVGVHP.[14].EKFA     KDE 96
query          9 YIDIGINLT.[20].VLQRAVGA.[2].KKFMVTGS.[5].KHAIGI.[8].YATVGVHP.[20].KTLA.[4].ETG 113
gi 34540566   11 LIDTHTHVY.[10].VILAAQEA.[2].IHLVMPNI.[6].RMQGVL.[7].SEAMGLHP.[14].RHEL     DTR 95
gi 15828485    3 YIDLHTHPF.[ 9].EIESWYSQ.[2].KKLFFVSC.[5].DQVIKA.[7].FPVLGTHP.[ 8].GEYL     AKK 79
gi 1176481     5 FIDTHCHFD.[10].SLQRAAQA.[2].GKIIVPAT.[5].ARVLAL.[7].YAALGLHP.[16].ALER     RPA 90
gi 17559024    6 LVDIGANLG.[10].VLDRAKQA.[2].SKIMVTGT.[5].HECADL.[8].YFTAGVHP.[14].KKLQ     ENP 90
gi 34394881    8 LIDIAVNFT.[20].VLARAWAA.[2].DRIIVTGG.[5].REALEI.[7].FCTVGVHP.[18].LLAL     AKE 105
gi 15639963    3 IFDTHAHIG.[11].VVQEARRA.[2].TRIMSICN.[5].AAVYET.[7].YHAVGVSP.[14].QKSL     QLP 87
gi 23473145   48 GVDSHAHLD.[10].ILTRAREA.[2].SHIGQVFL.[5].AQNRHL.[7].FFILGLHP.[15].QAFA     TDP 132
gi 31544922    6 LYDTHCHVN.[ 9].ELLKAFDQ.[2].IYINVVGT.[5].ILANDI.[7].SACIAVHP.[15].EQML     STP 89

Feature 1                                                      #                         #
1J6O_A        97 .[2].VAIGETGLDFF.[8].QKRVFVEQIELAG.[4].PLVVHIR.[3].SEAYEILR.[7].RGVIHAFS.[4].WAK 174
query        114 .[2].VAFGEIGLDYD.[9].QLKYFEAQLEIAV.[4].PLFLHSR.[3].EDFERLLT.[6].RGLVHSFT.[4].EMQ 193
gi 34540566   96 .[2].VAIGEIGIDLY.[8].QVEAFLTQIEWSM.[4].PIIIHSR.[3].DVVFACLN.[7].RGVFHSFS.[4].DLR 173
gi 15828485   80 .[5].VAIGEIGLDYH.[9].QIESFISQLDVAL.[4].PVIIHCR.[3].DDIYEILS.[7].QIIFHTFS.[4].WAQ 161
gi 1176481    91 .[2].VAVGEIGLDLF.[8].QQWLLDEQLKLAK.[4].PVILHSR.[3].DKLAMHLK.[6].TGVVHGFS.[4].QAE 167
gi 17559024   91 .[2].VAVGECGLDFN.[8].QKEVFAKQVDMAV.[4].PLFIHER.[3].EDMVKILT.[7].PAVIHCFT.[4].EAK 168
gi 34394881  106 .[7].VAVGECGLDYD.[9].QKKYFKKQFELAE.[4].PMFLHMR.[3].EDFCEIVS.[7].GGVTHSFT.[4].DRD 189
gi 15639963   88 .[2].VALGETGLDYC.[8].QIGLFITQLDIAS.[4].PVIIHNR.[3].QDILDILS.[6].GGVFHCYS.[4].YAR 164
gi 23473145  133 .[2].KAVGEIGLDYY.[8].QQKTFVMQLRMAV.[4].PVVIHSR.[3].EDTVRLLE.[7].PLLWHCFG.[4].LAE 210
gi 31544922   90 .[4].VGIGECGLDFY.[8].QYQFLDMHLELAN.[4].PLMLHIR.[3].KELVEYLN.[6].PIIFHCFS.[4].TYK 168

Feature 1                                                 #
1J6O_A       175 RF.[3].GFLLGIGGPVTY.[ 7].EVVKRVG.[3].IVLETDCPFLPP.[ 7].NEPKYLKYVVETISQV.[7].VDEA 254
query        194 RL.[3].GFDIGVNGCSMK.[ 5].AVVKQIP.[3].LQIETDGPWCEM.[34].NEPATIPHVAHAIAKI.[7].VCDA 298
gi 34540566  174 RA.[4].HFYIGINGTVTF.[ 6].ALLPLIP.[3].LLLETDSPYLAP.[ 7].NEPAYLVHTATFIAHI.[7].LAEK 253
gi 15828485  162 KL.[3].NFYLSFSGVLTF.[ 7].EVLKITP.[3].IFFETDSPYLAP.[ 7].NKSTYVKEVYKLASEI.[7].LIAQ 241
gi 1176481   168 RF.[3].GYKIGVGGTITY.[ 7].DVIAKLP.[3].LLLETDAPDMPL.[ 7].NRPEQAARVFAVLCEL.[7].IAQA 247
gi 17559024  169 KY.[3].GFYIGLTGFLWK.[10].LRSGEIP.[3].LVLETDAPYMYP.[30].NEPCSLAAVCELVAAF.[7].VAKI 274
gi 34394881  190 KL.[4].KMFIGINGCSLK.[ 5].EVLQGIP.[3].MMIETDSPYCDI.[31].NEPCLVRQVLEVVAGC.[8].LSKT 293
gi 15639963  165 MA.[3].PVYFSFAGNLTY.[ 7].ETVLALP.[3].ILVESESPFMSP.[ 7].NRPAHTVETVEFMAEL.[7].LADQ 244
gi 23473145  211 RI.[3].GWHVSVPGPVTY.[ 7].EALSVIP.[3].LMLETDCPYLTP.[ 7].NEPALAAFTGACVARC.[7].LWTR 290
gi 31544922  169 LL.[7].VRFYSIPGVVTF.[ 7].EVIPLIK.[3].LLVETDAPFLTP.[ 7].NNSLYLKYTIEKIAEL.[7].IKQL 252

Feature 1
1J6O_A       255 TTENARRIFL 264
query        299 AWKNSVKMFG 308
gi 34540566  254 TARNACRFFG 263
gi 15828485  242 VEQNVKKVFG 251
gi 1176481   248 LLNNTYTLFN 257
gi 17559024  275 TTENAKKVYK 284
gi 34394881  294 LYHNTCRLFF 303
gi 15639963  245 LWKNSCACFH 254
gi 23473145  291 CGNNARAFFG 300
gi 31544922  253 TFDNAYNLFK 262
```

Figure S3A

JGI 149183 (Nuc2)

```
Feature 1
1J6O_A       13  .[19].VISSFEEN.[2].EFVVNVGV.[5].KKSLDL.[7].FCSVGVHP.[14].EKFAKDE.[2].VAIGETGLDFF 109
query        22  .[29].VLDRALAA.[2].EKVMLTGM.[5].FVNIAI.[6].KITIGVHP.[22].RTMEQEP.[3].AAFGELGLDYD 138
gi 34540566  11  .[19].VILAAQEA.[2].IHLVMPNI.[6].RMQGVL.[7].SEAMGLHP.[14].RHELDTR.[2].VAIGEIGIDLY 108
gi 23022333   4  .[19].VIKKAYDS.[2].SYILNAAA.[5].LETVSL.[7].YGAVGVHP.[14].KDFAKEE.[2].VAIGEIGLDYY 100
gi 15828485   3  .[18].EIESWYSQ.[2].KKLFFVSC.[5].DQVIKA.[7].FPVLGTHP.[ 8].GEYLAKK.[5].VAIGEIGLDYH 95
gi 1176481    5  .[19].SLQRAAQA.[2].GKIIVPAT.[5].ARVLAL.[7].YAALGLHP.[16].ALERRPA.[2].VAVGEIGLDLF 103
gi 17559024   6  .[19].VLDRAKQA.[2].SKIMVIGT.[5].HECADL.[8].YFTAGVHP.[14].KKLQENP.[2].VAVGECGLDFN 103
gi 34394881   8  .[29].VLARAWAA.[2].DRIIVTGG.[5].REALEI.[7].FCTVGVHP.[18].LLALAKE.[7].VAVGECGLDYD 123
gi 23473145  48  .[19].ILTRAREA.[2].SHIGQVFL.[5].AQNRHL.[7].FFILGLHP.[15].QAFATDP.[2].KAVGEIGLDYY 145
gi 31544922   6  .[18].ELLKAFDQ.[2].IYINVVGT.[5].ILANDI.[7].SACIAVHP.[15].EQMLSTP.[4].VGIGECGLDFY 104

Feature 1                               ‡                             ‡
1J6O_A      110 .[8].QKRVFVEQIELAG.[4].PLVVHIR.[3].SEAYEILR.[7].RGVIHAFS.[4].WAKKF.[3].GFLLGIG 186
query       139 .[9].QIRVFRRQLDMIV.[6].PLFLHCR.[3].EDFVSILE.[9].SGLVHSFV.[4].QMQKL.[3].GLHVSVN 220
gi 34540566 109 .[8].QVEAFLTQIEWSM.[4].PIIIHSR.[3].DVVFACLN.[7].RGVFHSFS.[4].DLRRA.[4].HFYIGIN 186
gi 23022333 101 .[8].QKLWFARQINLAK.[4].PIIVHDR.[3].KDVLDIVK.[7].GGVFHCYS.[4].MLKDV.[3].NFYISVG 177
gi 15828485  96 .[9].QIESFISQLDVAL.[4].PVIIHCR.[3].DDIYEILS.[7].QIIFHTFS.[4].WAQKL.[3].NFYLSFS 173
gi 1176481  104 .[8].QQWLLDEQLKLAK.[4].PVILHSR.[3].DKLAMHLK.[6].TGVVHGFS.[4].QAERF.[3].GYKIGVG 179
gi 17559024 104 .[8].QKEVFAKQVDMAV.[4].PLFIHER.[3].EDMVKILT.[7].PAVIHCFT.[4].EAKKY.[3].GFYIGLT 180
gi 34394881 124 .[9].QKKYFKKQFELAE.[4].PMFLHMR.[3].EDFCEIVS.[7].GGVTHSFT.[4].DRDKL.[4].KMFIGIN 202
gi 23473145 146 .[8].QQKTFVMQLRMAV.[4].PVVIHSR.[3].EDTVRLLE.[7].PLLWHCFG.[4].LAERI.[3].GWHVSVP 222
gi 31544922 105 .[8].QYQFLDMHLELAN.[4].PLMLHIR.[3].KELVEYLN.[6].PIIFHCFS.[4].TYKLL.[7].VRFYSIP 184

Feature 1                                           ‡
1J6O_A      187 .[1].PVTY.[ 7].EVVKRVG.[3].IVLETDCPF         LPP.[ 7].NEPKYLKYVVETISQV.[7].VDEATTEN 258
query       221      NFAF.[ 6].EMVRDVP.[3].LQVETDAPW.[29].FSL.[ 7].NESCNVEKVALVVSGL.[7].VAESAWGN 319
gi 34540566 187 .[1].TVTF.[ 6].ALLPLIP.[3].LLLETDSPY         LAP.[ 7].NEPAYLVHIATFIAHI.[7].LAEKTARN 257
gi 23022333 178 .[1].TLTF.[ 7].EVVERVP.[3].LLIETDCPY         LTP.[ 7].NDSSYVRLVAEKIAKI.[7].VAEITTNN 249
gi 15828485 174 .[1].VLTF.[ 7].EVLKITP.[3].IFFETDSPY         LAP.[ 7].NKSTYVKEVYKLASEI.[7].LIAQVEQN 245
gi 1176481  180 .[1].TITY.[ 7].DVIAKLP.[3].LLLETDAPD         MPL.[ 7].NRPEQAARVFAVLCEL.[7].IAQALLNN 251
gi 17559024 181 .[1].FLWK.[10].LRSGEIP.[3].LVLETDAPY         MYP.[30].NEPCSLAAVCELVAAF.[7].VAKITTEN 278
gi 34394881 203 .[1].CSLK.[ 5].EVLQGIP.[3].MMIETDSPY         CDI.[31].NEPCLVRQVLEVVAGC.[8].LSKTLYHN 297
gi 23473145 223 .[1].PVTY.[ 7].EALSVIP.[3].LMLETDCPY         LTP.[ 7].NEPALAAFTGACVARC.[7].LWTRCGNN 294
gi 31544922 185 .[1].VVTF.[ 7].EVIPLIK.[3].LLVETDAPF         LTP.[ 7].NNSLYLKYTIEKIAEL.[7].IKQLTFDN 256

Feature 1
1J6O_A      259 ARRIFL 264
query       320 SITMFF 325
gi 34540566 258 ACRFFG 263
gi 23022333 250 AKRLFG 255
gi 15828485 246 VKKVFG 251
gi 1176481  252 TYTLFN 257
gi 17559024 279 AKKVYK 284
gi 34394881 298 TCRLFF 303
gi 23473145 295 ARAFFG 300
gi 31544922 257 AYNLFK 262
```

Figure S3B
