# Supplementary figures and images for: A DNase from a Fungal Phytopathogen Is a Virulence Factor Likely Deployed as Counter Defense against Host-Secreted Extracellular DNA
Source: mBio. 2019 Mar 5;10(2):e02805-18. doi: 10.1128/mBio.02805-18 (PMC6401486; doi:10.1128/mBio.02805-18)

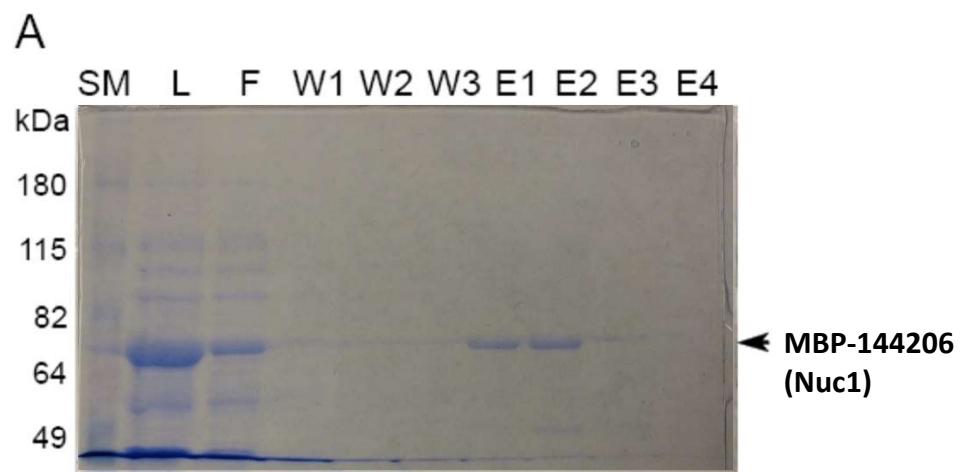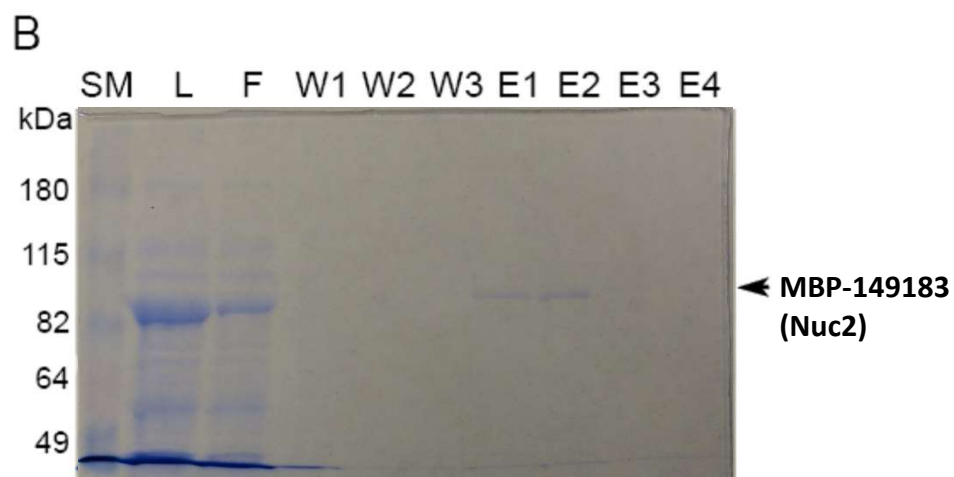

Figure S4

Supplement: FIG S4 [file mBio.02805-18-sf004.pdf]

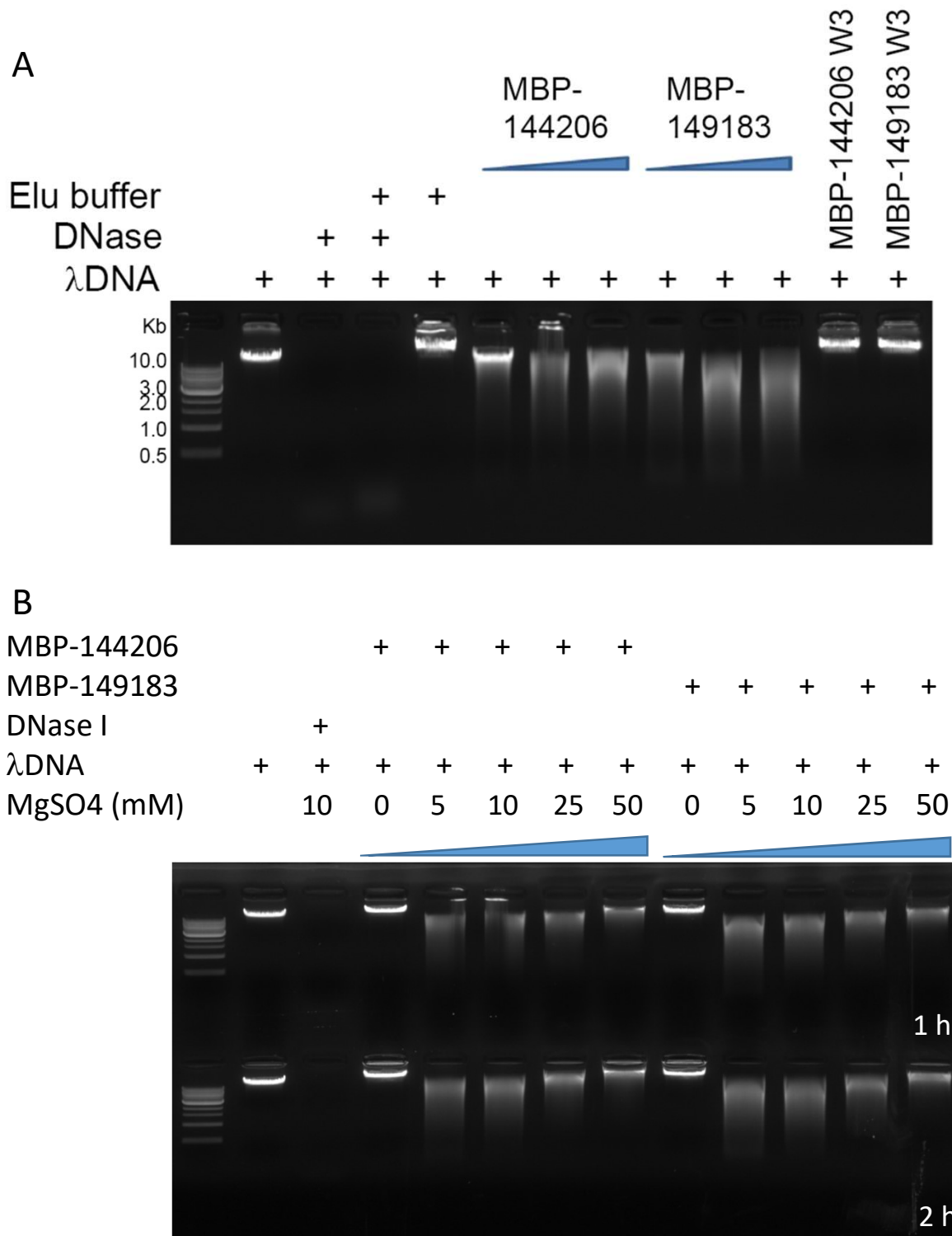

Figure S5

Supplement: FIG S5 [file mBio.02805-18-sf005.pdf]

**A**

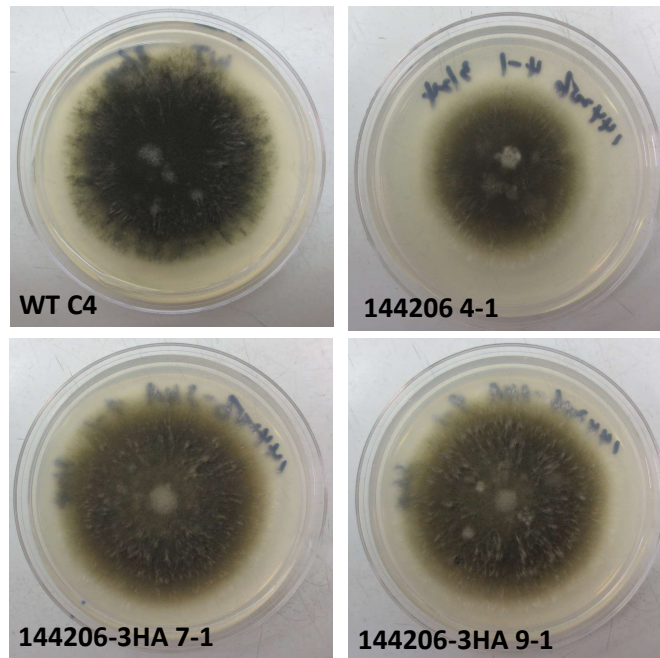

**B**

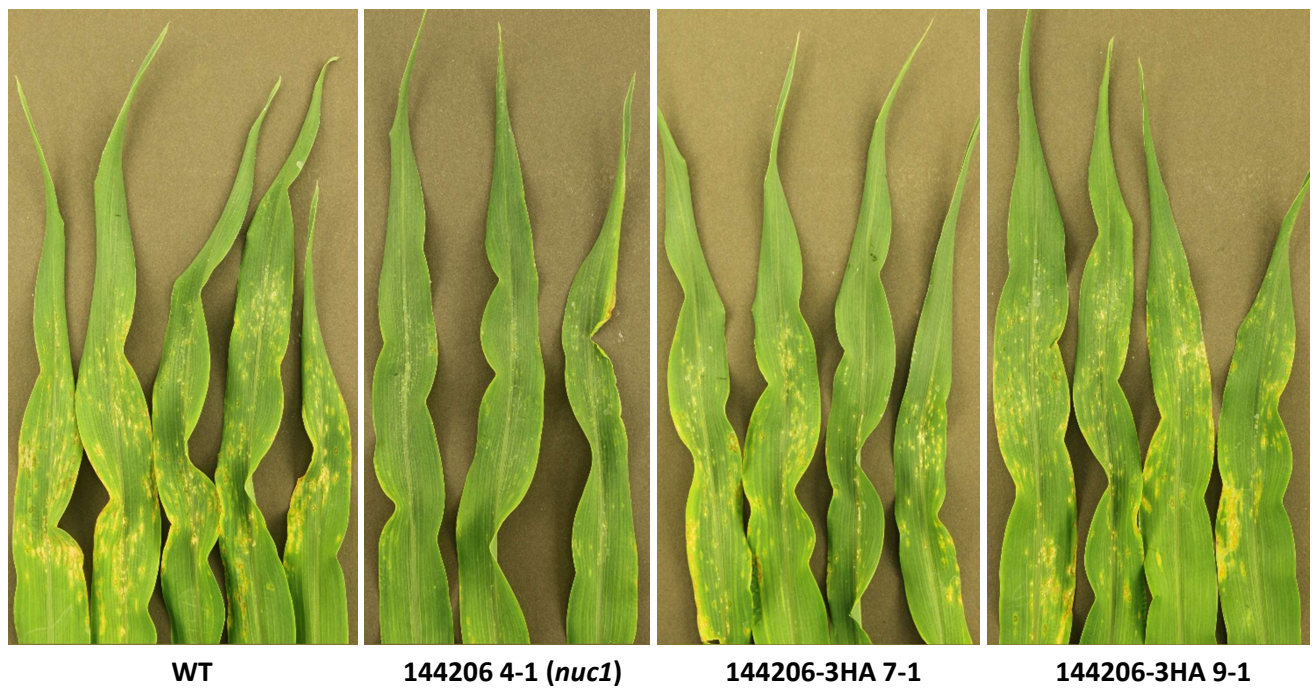

**Figure S7**

Supplement: FIG S7 [file mBio.02805-18-sf007.pdf]

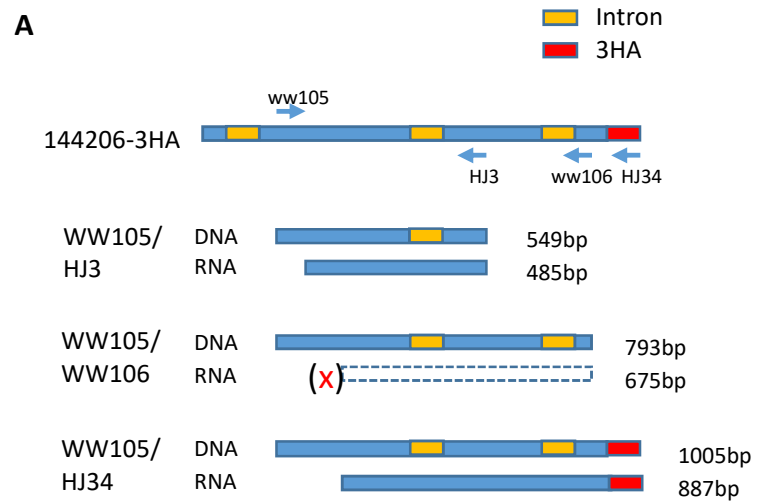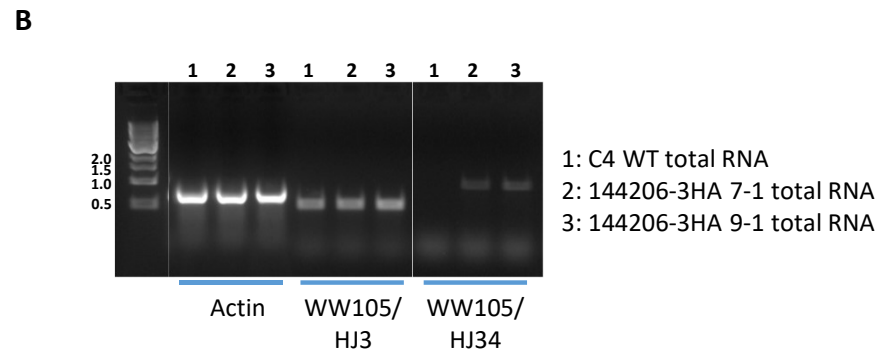

**Figure S8**

Supplement: FIG S8 [file mBio.02805-18-sf008.pdf]

**A**

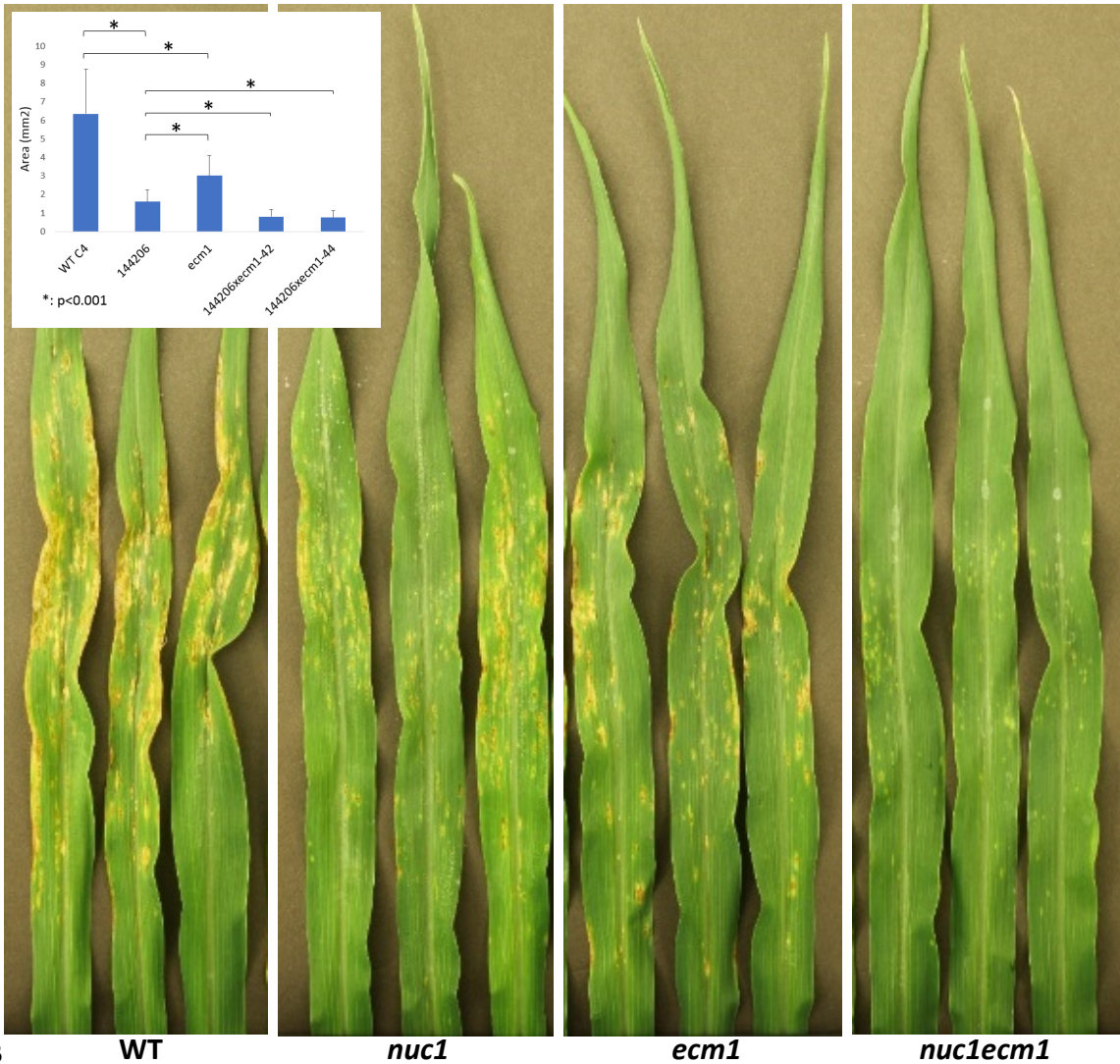

**B**

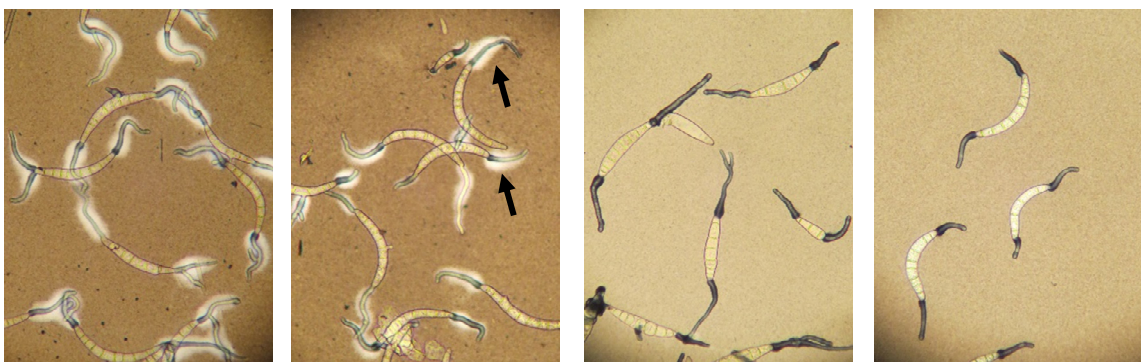

Figure S9

Supplement: FIG S9 [file mBio.02805-18-sf009.pdf]
