# Supplementary material for: A DNase from a Fungal Phytopathogen Is a Virulence Factor Likely Deployed as Counter Defense against Host-Secreted Extracellular DNA
Source: mBio. 2019 Mar 5;10(2):e02805-18. doi: 10.1128/mBio.02805-18 (PMC6401486; doi:10.1128/mBio.02805-18)
Supplement: TABLE S1 [file mBio.02805-18-st001.pdf]

Table S1. Primers

Name	Sequence	Purpose
WW91	GCCTTTATTGTCTTCGAGTCT	Forward primer, amplification of 5’ flank of 33717
WW92	tcctgtgtgaaattgttatccgctACTAGTCCTTGTCACCCATCT	Reverse primer, amplification of 5’ flank of 33717
WW93	gtcgtgactgggaaaaccctggcgCTGTTGACTTTTCTCTCTCCA	Forward primer, amplification of 3’ flank of 33717
WW94	ACCTCTATCCTTTCCTTTCCT	Reverse primer, amplification of 3’ flank of 33717
WW95	CTCTACATCACCTACGACGAG	Forward primer upstream of F1, to confirm insertion
WW96	GTCATCAAGAAGAGCATTGAG	Reverse primer downstream of F2, to confirm insertion
WW97	ACACTACAAAACAGCCCTACA	Forward primer, amplification of 33717 fragment
WW98	CTACGCACAATATCTTCAAGC	Reverse primer, amplification of 33717 fragment
WW99	GTTTGTCTTCGTCTTCTTCCT	Forward primer, amplification of 5’ flank of 144206
WW100	tcctgtgtgaaattgttatccgctGTGTCCCTAAACAGTTTTCGT	Reverse primer, amplification of 5’ flank of 144206
WW101	gtcgtgactgggaaaaccctggcgGCTACTTGGTCAAGTGTTGTC	Forward primer, amplification of 3’ flank of 144206
WW102	GTGATGAGGTACTCACGTGTT	Reverse primer, amplification of 3’ flank of 144206
WW103	GCTGTTGTATTACTGCTGTCC	Forward primer upstream of F1, to confirm insertion
WW104	TCAGTATAGGAACAGGTCAGG	Reverse primer downstream of F2, to confirm insertion
WW105	TACTGTAGGCGTACATCCTTG	Forward primer, amplification of 144206 fragment
WW106	TTCCAAGCACTACACTCTCAT	Reverse primer, amplification of 144206 fragment
WW107	TGAGACCACTCAGTAACGACT	Forward primer, amplification of 5’ flank of 149183
WW108	tcctgtgtgaaattgttatccgctTGTTGTGGTTAGAGGTACTGG	Reverse primer, amplification of 5’ flank of 149183
WW109	gtcgtgactgggaaaaccctggcgAGCTCACAGTTCCTGTATTGA	Forward primer, amplification of 3’ flank of 149183
WW110	ATGTACGGCGACACATATACT	Reverse primer, amplification of 3’ flank of 149183
WW111	TGTAAGCTGTACTGAGGGAAA	Forward primer upstream of F1, to confirm insertion
WW112	GACCGGAGACTACACTATCCT	Reverse primer downstream of F2, to confirm insertion
WW113	CTGTAACAGCAACAGCAAAG	Forward primer, amplification of 149183 fragment
WW114	CAACTCTCATTACGCTCCTTA	Reverse primer, amplification of 149183 fragment
WW115	GGTAGGAGCAAAAGGTAGAAT	Forward primer, amplification of 5’ flank of 83474
WW116	tcctgtgtgaaattgttatccgctCTCTCATGCAATTTCTACACC	Reverse primer, amplification of 5’ flank of 83474
WW117	gtcgtgactgggaaaaccctggcgGGTTTAGAGTGTGTGTCCAAC	Forward primer, amplification of 3’ flank of 83474
WW118	CCGACATGTTAGTCCACTATG	Reverse primer, amplification of 3’ flank of 83474
WW119	ATATCTGTTGCTACCCCAACT	Forward primer upstream of F1, to confirm insertion
WW120	AGTACCACCACCATGTCTGA	Reverse primer downstream of F2, to confirm insertion
WW121	TGTGATACTGACCCCGTATAG	Forward primer, amplification of 83474 fragment
WW122	CGAACTCGTTATAGTCACCTG	Reverse primer, amplification of 83474 fragment

WW123	CCACACCATACCTTACATCTC	Forward primer, amplification of 5’ flank of 122478
WW124	tcctgtgtgaaattgttatccgctGCCTTTACAGTAAGGACCAGT	Reverse primer, amplification of 5’ flank of 122478
WW125	gtcgtgactgggaaaaccctggcgGACAGCTGTAAGTGGAGTCAC	Forward primer, amplification of 3’ flank of 122478
WW126	GTCCAGTGATTTACCCTTGTA	Reverse primer, amplification of 3’ flank of 122478
WW127	ATCAGCACTAAACATCTGCTG	Forward primer upstream of F1, to confirm insertion
WW128	TTTACGAGTAAGAACGGTACG	Reverse primer downstream of F2, to confirm insertion
WW129	GCTGGGTAAGTCTCGATTAGT	Forward primer, amplification of 122478 fragment
WW130	GTCTTGAACTGTGCGTAAAAC	Reverse primer, amplification of 122478 fragment
M13R	AGCGGATAACAATTTCACACAGGA	Reverse primer, amplification of HygB
M13F	CGCCAGGGTTTTCCCAGTCACGAC	Forward primer, amplification of HygB
PtrpC	GGTCGTTCACTTACCTTGCTTG	Reverse primer, to confirm insertion
TtrpCend	GTGAATGCTCCGTAACACCCAATAC	Forward primer, to confirm insertion
WW269	GCTACTTGGTCAAGTGTTGTC	Forward primer, amplification of F2
WW270	aagatcactggaacaactggcatgGTGATGAGGTACTCACGTGTT	Reverse primer, amplification of F2
WW271	gcacaggtacacttgtttagaggtACTTACTCCTGACCTGTTCCT	Forward primer, amplification of F3
WW272	ATCTGCTCTCACATTGGACT	Reverse primer, amplification of F3
WW273	TCGGTTCATAGTCAATCAGTC	Reverse primer downstream of F3, to confirm complementation
DW69	CATGCCAGTTGTTCCAGTG	Forward primer, amplification of nptII
DW70	ACCTCTAAACAAGTGTACCTG	Reverse primer, amplification of nptII
HJ10	cg gaa ttc ATG GTA ACA GGA TCT GAT CTC	Forward primer, amplification of 144206
HJ11	aaaa ctc gag TCA GTG TTG GTA AAC ATA CCA	Reverse primer, amplification of 144206
HJ12	cg gaa ttc ATG TTG ACA GGC ATG TAC G	Forward primer, amplification of 149183
HJ8	gtg gc ggcc gc TCA CGC ACT CGT TGA TGC	Reverse primer, amplification of 149183
