# Supplementary material for: A DNase from a Fungal Phytopathogen Is a Virulence Factor Likely Deployed as Counter Defense against Host-Secreted Extracellular DNA
Source: mBio. 2019 Mar 5;10(2):e02805-18. doi: 10.1128/mBio.02805-18 (PMC6401486; doi:10.1128/mBio.02805-18)
Supplement: FIG S6 [file mBio.02805-18-sf006.pdf]

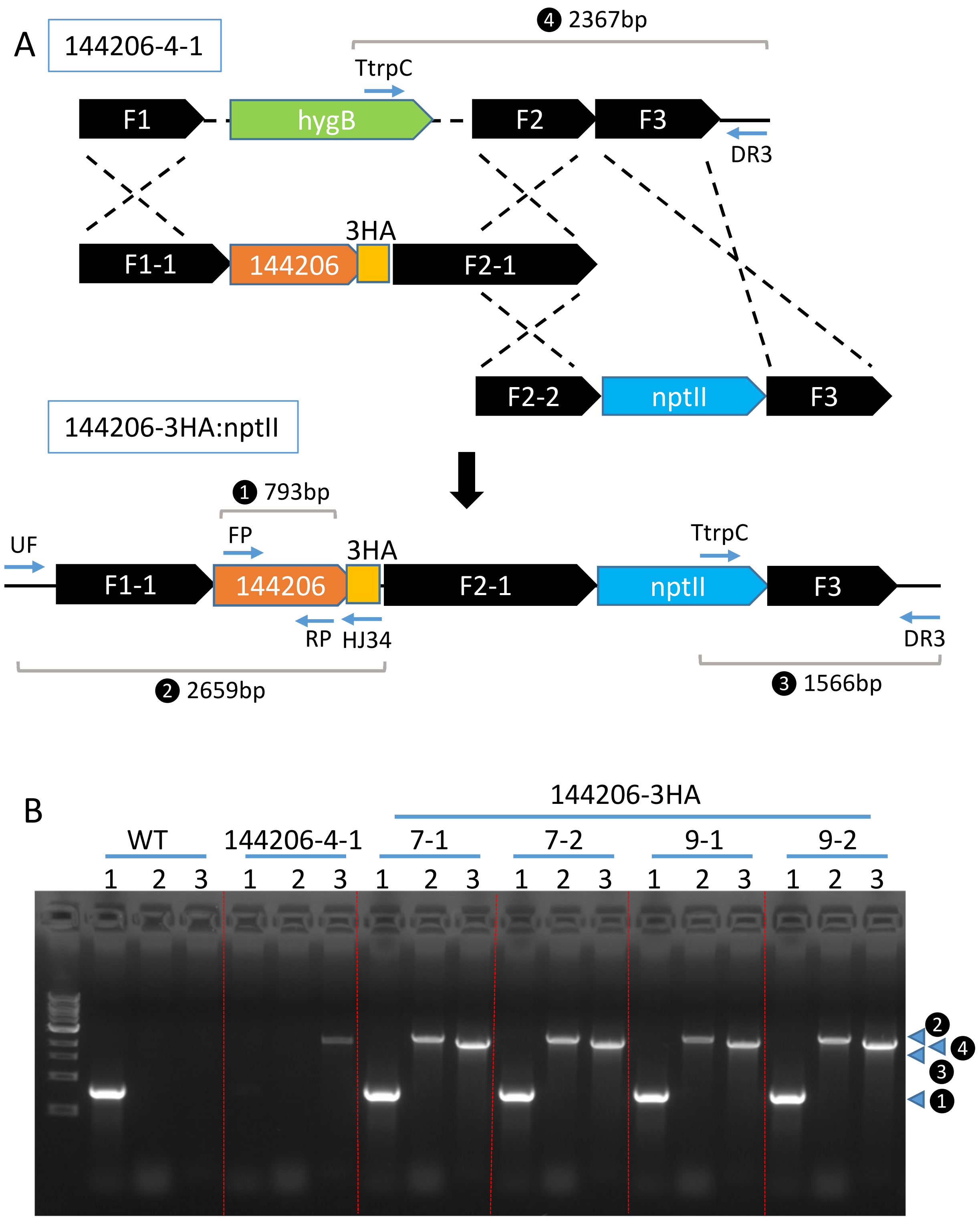

A
144206-4-1
❹ 2367bp
TtrpC
F1
hygB
F2
F3
DR3
3HA
F1-1
144206
F2-1
F2-2
nptII
F3
144206-3HA:nptII
❶ 793bp
FP
UF
F1-1
144206
3HA
F2-1
TtrpC
nptII
F3
RP
HJ34
DR3
❷ 2659bp
❸ 1566bp
B
144206-3HA
WT
144206-4-1
7-1
7-2
9-1
9-2
1 2 3 1 2 3 1 2 3 1 2 3 1 2 3 1 2 3
2
4
3
1

Figure S6
